# Comparative Transcriptomic Profiling of Yersinia enterocolitica O:3 and O:8 Reveals Major Expression Differences of Fitness- and Virulence-Relevant Genes Indicating Ecological Separation

**DOI:** 10.1128/mSystems.00239-18

**Published:** 2019-04-23

**Authors:** Carina Schmühl, Michael Beckstette, Ann Kathrin Heroven, Boyke Bunk, Cathrin Spröer, Alan McNally, Jörg Overmann, Petra Dersch

**Affiliations:** a Department of Molecular Infection Biology, Helmholtz Centre for Infection Research, Braunschweig, Germany; b Leibniz Institute DSMZ—German Collection of Microorganisms and Cell Cultures, Braunschweig, Germany; c Institute of Microbiology and Infection, University of Birmingham, Birmingham, United Kingdom; d Department of Microbiology, Technical University Braunschweig, Braunschweig, Germany; e German Center for Infection Research, Braunschweig, Germany; f Institute for Infectiology, Center for Molecular Biology of Inflammation, University of Münster, Münster, Germany; University of Pennsylvania

**Keywords:** Yersinia enterocolitica, YstA toxin, comparative transcriptomics, growth-phase control, temperature regulation

## Abstract

Yersinia enterocolitica is a zoonotic pathogen and an important cause of bacterial gastrointestinal infections in humans. Large-scale population genomic analyses revealed genetic and phenotypic diversity of this bacterial species, but little is known about the differences in the transcriptome organization, small RNA (sRNA) repertoire, and transcriptional output. Here, we present the first comparative high-resolution transcriptome analysis of Y. enterocolitica strains representing highly pathogenic phylogroup 2 (serotype O:8) and moderately pathogenic phylogroup 3 (serotype O:3) grown under four infection-relevant conditions. Our transcriptome sequencing (RNA-seq) approach revealed 1,299 and 1,076 transcriptional start sites and identified strain-specific sRNAs that could contribute to differential regulation among the phylogroups. Comparative transcriptomics further uncovered major gene expression differences, in particular, in the temperature-responsive regulon. Multiple virulence-relevant genes are differentially regulated between the two strains, supporting an ecological separation of phylogroups with certain niche-adapted properties. Strong upregulation of the *ystA* enterotoxin gene in combination with constitutive high expression of cell invasion factor InvA further showed that the toxicity of recent outbreak O:3 strains has increased. Overall, our report provides new insights into the specific transcriptome organization of phylogroups 2 and 3 and reveals gene expression differences contributing to the substantial phenotypic differences that exist between the lineages.

**IMPORTANCE** Yersinia enterocolitica is a major diarrheal pathogen and is associated with a large range of gut-associated diseases. Members of this species have evolved into different phylogroups with genotypic variations. We performed the first characterization of the Y. enterocolitica transcriptional landscape and tracked the consequences of the genomic variations between two different pathogenic phylogroups by comparing their RNA repertoire, promoter usage, and expression profiles under four different virulence-relevant conditions. Our analysis revealed major differences in the transcriptional outputs of the closely related strains, pointing to an ecological separation in which one is more adapted to an environmental lifestyle and the other to a mostly mammal-associated lifestyle. Moreover, a variety of pathoadaptive alterations, including alterations in acid resistance genes, colonization factors, and toxins, were identified which affect virulence and host specificity. This illustrates that comparative transcriptomics is an excellent approach to discover differences in the functional output from closely related genomes affecting niche adaptation and virulence, which cannot be directly inferred from DNA sequences.

## Abstract (precis)

Yersinia enterocolitica is a major diarrheal pathogen and is associated with a large range of gut-associated diseases. Members of this species have evolved into different phylogroups with genotypic variations. We performed the first characterization of the Y. enterocolitica transcriptional landscape and tracked the consequences of the genomic variations between two different pathogenic phylogroups by comparing their RNA repertoire, promoter usage, and expression profiles under four different virulence-relevant conditions. Our analysis revealed major differences in the transcriptional outputs of the closely related strains, pointing to an ecological separation in which one is more adapted to an environmental lifestyle and the other to a mostly mammal-associated lifestyle. Moreover, a variety of pathoadaptive alterations, including alterations in acid resistance genes, colonization factors, and toxins, were identified which affect virulence and host specificity. This illustrates that comparative transcriptomics is an excellent approach to discover differences in the functional output from closely related genomes affecting niche adaptation and virulence, which cannot be directly inferred from DNA sequences.

## INTRODUCTION

The enteric pathogen Yersinia enterocolitica is the most common Gram-negative zoonotic pathogen leading to human yersiniosis, representing a variety of gut-associated diseases ranging from enteritis, watery diarrhea, and mesenteric lymphadenitis to postinfectious extraintestinal sequelae such as reactive arthritis (1, 2). Yersiniosis is among the most common bacterial enteric diseases in the industrialized countries, with the highest burden of disease in children under 15 years of age (2–4). The species Y. enterocolitica constitutes a very diverse group of around 70 serotypes, of which only 11 are harmful to humans. The different serotypes were isolated from diarrhea patients, livestock, poultry, wild animals, insect vectors, food, and the environment. Among the isolated pathogenic strains are the highly mouse-virulent 1B/O:8 strains (YeO:8), recently classified into phylogroup 2 (5, 6). This bioserotype, in particular, YeO:8 strain 8081v, has been used to study the pathogenesis of Y. enterocolitica using mouse infection models. However, the most frequent cause of human yersiniosis (>90%) in Europe and Japan by far is Y. enterocolitica bioserotype O:3 (YeO:3), phylogroup 3, which is also frequently found in pigs and pork products (1, 3, 6, 7). It primarily originates from domestic pigs, in which the bacteria colonize the lymphoid tissue of the gut and oropharynx, mainly as asymptomatic carriers (8, 9). YeO:3 is less common in North America but has replaced YeO:8 (the most prevalent serotype in the 1990s) and is now the predominant serotype (10, 11). The reasons for its rising global relevance are largely unknown, but recent genomic comparisons and analyses of its host colonization properties revealed unique virulence properties and fitness determinants (5, 12).

The gene content and synteny of YeO:8 and YeO:3 strains are largely conserved. However, recent studies also demonstrated that there are considerable genomic differences (5, 13). YeO:3 does not contain certain pathogenicity factors of YeO:8, such as the high-pathogenicity island (HPI) involved in yersiniabactin (Ybt)-mediated iron uptake and the chromosomally encoded *ysa* type III secretion system (T3SS). Instead, it has evolved an alternative set of virulence-associated traits, including a RtxA-like toxin, two putative invasion-associated genes, two clusters of putative β-fimbriae, a dual-function insecticidal toxin, and another distinct *ysp* T3SS. The *ysp* T3SS is homologous to the *Salmonella* pathogenicity island 2 (SPI-2) T3SS but lacks functional parts; i.e., there are no effector genes linked to the T3SS gene cluster (13). Moreover, the *rtxA* toxin gene of the Rtx cluster is intact, but the secretion genes are disrupted, leaving the involvement of *ysp* and *rtx* genes in pathogenicity unclear. Moreover, the pYV plasmids are more divergent than the corresponding genome sequences, and the clusters for the lipopolysaccharide (LPS) outer core and the O-antigen are differently organized. The distinct virulence traits trigger different cytokine profiles shown by primary human, porcine, and murine macrophages. YeO:3 promotes a significant lower level of production of interleukin-8 (IL-8) but a considerably higher level of secretion of IL-10 (14). It is likely that this contributes to inhibiting inflammation and immunopathological changes and to favoring long-term persistence without severe clinical manifestations.

Genomic variations of YeO:3 that streamline the physiology and metabolism and increase the overall fitness of the bacteria with respect to their lifestyle are also likely to contribute to their worldwide success. For instance, YeO:3 strains, but not YeO:8 strains, possess the *aga* operon that allows them to grow on *N*-acetylgalactosamine (GalNAc) (13, 15). As GalNAc is the major amino sugar of porcine mucin, this metabolic trait may represent an important virulence-relevant fitness factor reflecting the adaptation of YeO:3 to its preferred reservoir host, the pig. In addition, several genomic islands as well as the spectra and numbers of prophages and insertion (IS) elements are different between YeO:3 and YeO:8. A wide variety of IS families (IS*3*, IS*4*, and IS*200*) dominate in YeO:8, whereas numerous ISYen1/IS*1667* and ISYen2 elements are found in YeO:3.

It is assumed that the genetic diversity of both serotypes leads to serotype-specific colonization and host-specific immune defense properties with different clinical outcomes (14), but how the serotype-specific characteristics impact pathogenicity in their preferred hosts is largely unclear. A comparative study of human-, pig-, and food-derived YeO:3 isolates with YeO:8 revealed that identical colonization factors participate in host cell binding and invasion and yet that small genetic variations lead to profound changes in their expression patterns (12, 16). An insertion of an ISYen1/IS*166*7 element caused a high level of constitutive expression of the primary cell binding and invasion factor InvA, and a base pair substitution resulted in the synthesis of a stable variant of the InvA regulator RovA in YeO:3. Both changes have a significant effect upon host cell invasion and virulence in mice and pigs (12, 16).

To gain insight into the dimension of the transcriptional variability that correlates with the genomic and phenotypic differences of the O:3 and O:8 serotypes, we used a comparative transcriptome sequencing (RNA-seq)-based approach to identify serotype/isolate-specific differences in the transcriptome under infection-relevant conditions. This strategy allowed us to obtain the first in-depth single-nucleotide resolution transcriptome of Y. enterocolitica (including genome-wide promoter maps and the noncoding RNA [ncRNA] repertoire), enabled us to reveal major differences in the temperature- and growth-phase-dependent expression profiles, and led to the discovery of changes that modulate transcript levels of important virulence-relevant traits.

## RESULTS AND DISCUSSION

### Comparative RNA-seq of Y. enterocolitica O:8 and O:3.

In order to obtain high-resolution transcription profiles and identify transcripts that were differentially expressed between serotype O:8 and O:3 strains, we built upon the transcriptome of YeO:8 strain 8081v and YeO:3 strain Y1. Strain 8081v is a well-characterized representative of YeO:8. It is a widely distributed and highly virulent isolate and has played a pivotal role in the analysis of *Yersinia* infection. Many of its virulence factors have been characterized in detail, and our knowledge of virulence-relevant gene regulation and networks was mainly derived from this strain, from which a complete genome sequence is available (RefSeq accession no. NC_008800.1 [chromosome] and NC_008791 [virulence plasmid]) (17). YeO:3 strain Y1 is a recent isolate from an outbreak in Germany isolated from a patient stool. Its cell adhesion and invasion properties and survival in macrophages, induction of immune responses, and virulence in mouse and pig models have been characterized and compared with those of strain 8081v (12, 14, 16). To allow precise mapping of the RNA sequencing reads, the genome of strain Y1 was sequenced *de novo*. Data were assembled into two circular replicons, one consisting of 4,522,295 bp for the genome (GenBank accession no. CP030980) and the other consisting of 72,411 bp for the pYVO:3 plasmid (GenBank accession no. CP030981), and both were annotated and used for transcriptome profiling. A sequence comparison with other available YeO:3 strains revealed average nucleotide identities of 99.9% to YeO:3 1203 and 99.83% to YeO:3 Y11 (see Fig. S1 in the supplemental material), making it a perfectly representative strain of highly clonal phylogroup 3.

10.1128/mSystems.00239-18.1 FIG S1 Average nucleotide identity of YeO:3 strain Y1 with other Y. enterocolitica and Y. pseudotuberculosis strains. The nucleotide sequence of YeO:3 strain Y1 was compared with the sequences of Y. enterocolitica serotype O:3 strains Y11 and 1203 and of Y. pseudotuberculosis strains YPIII and IP32953. The average nucleotide identities with other strains are indicated in percentages and were determined using pyANI software (https://github.com/widdowquinn/pyani) and MUMmer software (S. Kurtz, A. Phillippy, A. L. Delcher, M. Smoot, et al., Genome Biol 5:R12, 2004). Download FIG S1, TIF file, 1.6 MB. Copyright © 2019 Schmühl et al. 2019 Schmühl et al. This content is distributed under the terms of the Creative Commons Attribution 4.0 International license.

**FIG 1:**
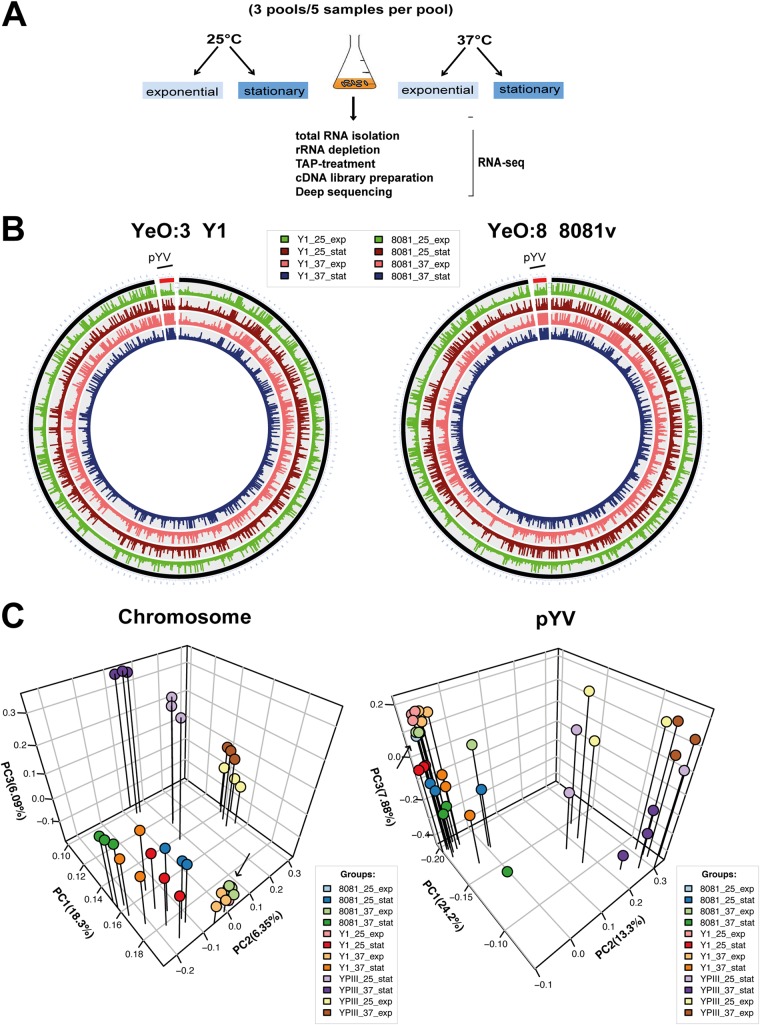
Comparative RNA-seq workflow and global reports. (A) For comparative *in vitro* RNA-seq analysis, Y. enterocolitica serotype O:3 strain Y1 and serotype O:8 strain 8081v were grown in LB to the exponential or stationary phase at 25°C or 37°C. Total RNA was isolated from bacterial cultures, processed for preparation of strand-specific barcoded cDNA libraries, and sequenced. cDNA reads were separated *in silico* by mapping to the 8081v and Y1 genomes. TAP, tobacco acid pyrophosphatase. (B) Circos plot visualizing replicate mean averaged RPKM (reads per kilobase transcript length per million mapped reads) and normalized expression values representing *in vitro* RNA-seq data determined for the Y. enterocolitica 8081v chromosome (NC_008791.1) and its virulence plasmid (pYVO:8; NC_008800.1) and the Y. enterocolitica Y1 chromosome (CP030980) and its virulence plasmid (pYVO:3; CP030981). 25_exp, exponential phase 25°C; 37_exp, exponential phase 37°C; 25_stat, stationary phase 25°C; 37_stat, stationary phase 37°C. (C) Principal-component analysis of mean centered and scaled log2-transformed RPKM counts of the RNA-seq data for the Y. pseudotuberculosis YPIII (19), Y. enterocolitica 8081v, and the Y. enterocolitica Y1 genome sequences and their virulence plasmids. The analysis is based on the 2,802 core genes and 51 plasmid core genes. The arrow indicates data points for 25_exp, which are hidden under other data points.

In order to obtain a comprehensive image of the primary transcriptome, we used rRNA-depleted total RNA of YeO:8 strain 8081v and YeO:3 strain Y1 grown to the exponential or stationary phase at 25°C or 37°C, conditions resembling alterations in temperatures and nutrient limitations encountered in the initial or later/ongoing stages of infection (Fig. 1A). A global RNA-seq approach was employed by comparing mapped sequence reads from different strand-specific barcoded cDNA libraries of three independent biological replicates of five pooled cultures for each growth condition to catalog the transcripts for a detailed gene map. Between 0.7 million and 3.0 million uniquely mapped sequence reads were generated from each library and mapped to the Y. enterocolitica 8081v genome (RefSeq accession no. NC_008791.1 [chromosome] and NC_008800.1 [virulence plasmid pYVO:8]) or to the Y. enterocolitica Y1 genome (RefSeq accession no. CP030980 [chromosome] and CP030981 [virulence plasmid pYVO:3]) (see Data Set S1-1 in the supplemental material). These represent sufficient coverage and robust representation of the Y. enterocolitica transcriptome under each of the four conditions (Fig. 1B; see also Data Set S1-1). The global gene expression profiles of the two strains were distinct, and the three biological triplicates clustered together (Fig. 1C).

10.1128/mSystems.00239-18.7 DATA SET S1 Mapping statistics, transcriptional start site (TSS), putative riboswitches and RNA thermometers, and noncoding RNAs of Y. enterocolitica 8081 and Y1. Data sheet S1-1, mapping statistics; data sheet S1-2, Y. enterocolitica 8081 transcriptional start site (TSS) and the 5′-UTR repertoires (this table lists all identified mTSSs and lmTSSs of YeO:8 8081v); data sheet S1-3, Y. enterocolitica Y1 transcriptional start site (TSS) maps and the 5′-UTR repertoires (this table lists all identified mTSSs and lmTSSs of YeO:3 Y1); data sheet S1-4, comparison of the TSSs of Y. enterocolitica 8081v and Y1 (this table lists compares all identified mTSSs and lmTSSs of YeO:8 8081v and YeO:3 Y1); data sheets S1-6 and S1-7, identification of putative riboswitches and RNA thermometers of *Y. entercolitica* 8081 and Y1 by the use of the RibEx riboswitch explorer and the Rfam database (this table lists the predicted riboswitches and RNA thermometers of YeO:8 strain 8081v and of YeO:3 strain Y1); data sheet S1-8, identification and expression profiling of sRNA candidates in Y. enterocolitica 8081 and Y1 (this table lists antisense RNAs of YeO:8 strain 8081v and YeO:3 strain Y1). Download Data Set S1, XLSX file, 0.8 MB. Copyright © 2019 Schmühl et al. 2019 Schmühl et al. This content is distributed under the terms of the Creative Commons Attribution 4.0 International license.

### The transcriptional landscape of Y. enterocolitica.

**FIG 2:**
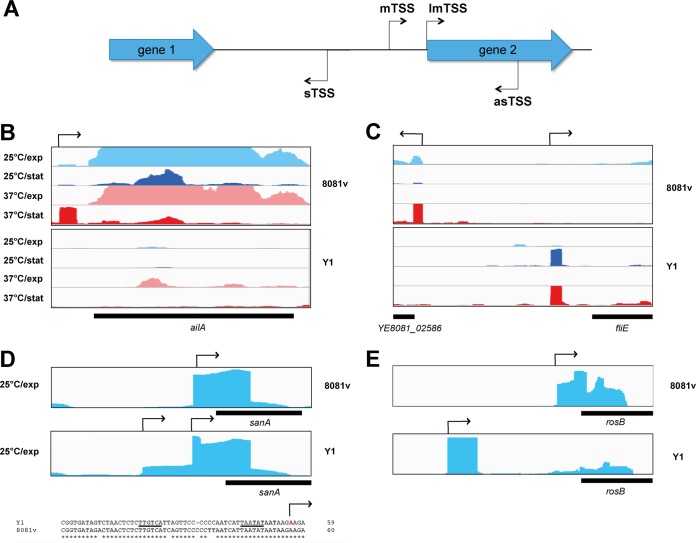
Comparative analysis of mRNA transcriptional start sites (TSSs) of Y1 and 8081v. (A) Schematic overview of the identified TSS: mTSS for mRNAs, lmTSS for leaderless transcripts, and sTSS and asTSS for the start site of small *trans*-acting regulatory RNAs and antisense RNAs. (B to E) Visualization of RNA-seq-based cDNA sequencing reads mapped to the 8081v and Y1 *ail* (B), *fliE* (C), *sanA* (D), and *rosB* (E) gene loci using the Integrative Genomics Viewer (IGV) genome browser. The transcriptional start sites are indicated by broken arrows. The promoter region of the *sanA* gene is given in panel C. The −10 and −35 regions of the upstream promoter of the *sanA* gene are underlined. The TSS identified in YeO:3 Y1 strain is indicated in red.

To generate a comprehensive map of transcriptional start sites (TSSs) for Y. enterocolitica, we adopted a method in which four virulence-relevant growth conditions were used to monitor transcription activation (18, 19). We categorized the identified transcription start using the terms “mTSSs” for mRNAs, “lmTSSs” for leader-less transcripts, and “sTSSs” and “asTSSs” for the start site of small *trans*-acting regulatory RNAs and antisense RNAs, respectively (Fig. 2A). We identified 1,299 mTSSs located upstream of the coding sequence for YeO:8 strain 8081v and 1,076 mTSS for YeO:3 Y1 (Data Sets S1-2 and S1-3). This revealed the global set of active gene promoters across the chromosome and the virulence plasmid of the species Y. enterocolitica for the first time. More TSSs could possibly be added to the maps under special growth or environmental stress conditions (e.g., acidic pH, high osmolarity, low oxygen), similarly to what has been shown for Salmonella enterica, in which the number of identified TSSs was increased from approximately 1,800 to 3,300 when 21 conditions were applied (20, 21).

To validate the mTSSs, mTSSs of published genes/operons of Y. enterocolitica were compared with the mTSSs predicted by the RNA-seq analysis. The vast majority of the small set of previously identified mTSSs consisted of identical examples. As an example, all mTSSs that had been identified previously within the *ompF*, *ompX*, and *ybtA* upstream region in Y. enterocolitica (22, 23) were also detected in our RNA-seq approach. We further determined the level of conservation of transcriptional organization between YeO:8 and YeO:3 by identifying not only common but also strain-specific TSSs. The location of 882 mTSS identified for strain 8081v was conserved in Y1 (70% to 80% identity), but 417 and 194 mTSSs were solely identified in YeO:8 8081v and in YeO:3 Y1, respectively (Data Sets S1-2 and S1-3).

Many of the strain-specific TSSs were from strain-specific hypothetical proteins, phages, and mobile elements, but several unique TSSs were from genes that are not expressed or are expressed only at very low levels in the other strain. As shown in Fig. 2B, the gene for the attachment and invasion locus (*ailA*) was significantly expressed in YeO:8 8081v with a transcript starting 59 nucleotides (nt) upstream of its translational start site but was not expressed or was expressed only at very low levels in YeO:3 Y1. In contrast, the *fliE* gene was expressed only in Y1 (Fig. 2C). Moreover, for the expression of some genes, different promoters are used. For instance, two start sites at position 80 and position 35 upstream of the translational start site were observed for the *sanA* gene in YeO:3 Y1, but only the proximal TSS was used in YeO:8 8081v (Fig. 2D). Pairwise comparison of the promoter region revealed four nucleotide exchanges and one nucleotide insertion upstream of the more distal TSS in the YeO:8 8081v, increasing the space between the putative −35 and −10 promoter regions. It is very likely that this results in a drastic loss of RNA polymerase binding and promoter activity. For some genes, such as *rosB*, different promoters were used under all tested growth conditions, leading to 5′ untranslated regions (5′-UTRs) with significantly different lengths (247 nt in Y1 and 46 nt 8081v; Fig. 2E). This indicated that there are many differences in the general promoter pattern and that the differences could be based either on certain variations on the DNA sequence level of the regulatory region or on the distinct expression/function of regulatory proteins.

**FIG 3:**
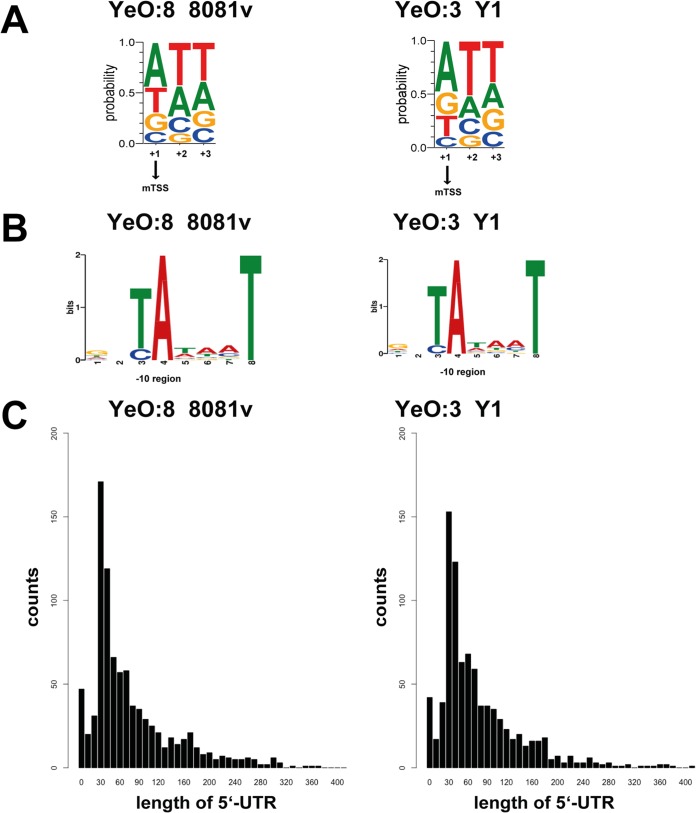
Global identification of mRNA transcriptional start sites (TSSs). (A) Sequence conservation at the TSSs. Sequence logo data were computed from 1,299 unaligned TSSs of YeO:8 strain 8081v and 1,076 unaligned TSS regions (TSS is located at position +1) showing nucleotide conservation around the TSSs. The initial nucleotides of transcripts (position +1 to position +3) are dominated by purines. (B) Detected conserved sequence motifs in the −10 promoter region (Pribnow box). (C) The distributions and frequencies of the lengths of 5′-UTRs are given for all mRNAs of Y1 and 8081v, which start upstream of the annotated TSS. More than 40% of all 5′-UTRs were 20 to 60 nt in length.

To detect conserved promoter sequence motifs for canonical RNA polymerase (RpoD) binding sites, we used MEME (24) analysis within the −10 region (positions −15 to −3) and the −35 region (positions −45 to −25). Alignment of all identified TSSs and promoter sequences revealed that adenine is the most common initiating nucleotide (A > 40%) and TAtaaT (highly conserved nucleotides are uppercase and less conserved nucleotides are lowercase) is the detected −10 Pribnow box region (Fig. 3A and B), characteristics which are very similar between YeO:3 and YeO:8 and homologous to those of Y. pseudotuberculosis (18, 19). In contrast to Y. pseudotuberculosis (with a −35 region of TTGC/A), but, similarly to some other pathogens (25–28), no strong canonical −35 region could be identified, even when only 115 promoters with a high expression rate (>100 reads) were included in the analysis.

The majority of transcripts of YeO:3 Y1 and YeO:8 8081v possessed untranslated regions (5′-UTRs) that were 20 to 60 nt in length (Fig. 3C; see also Data Sets S1-2, S1-3, and S1-4), highly similarly to other bacteria (21, 26, 29, 30). However, 141 and 182 5′-UTRs were longer than 150 nt and subsets of 13 and 16 mRNAs had 5′-UTRs longer than 300 nt in YeO:3 Y1 and YeO:8 8081v, respectively (Data Sets S1-2, S1-3, S1-4, S1-5, and S1-6). These long 5′-UTRs could include putative *cis*-regulatory RNA elements, such as RNA riboswitches and RNA thermometers, known to control transcription, translation initiation, and stability of mRNAs (31-34). A more detailed inspection using RibEx riboswitch explorer (35) and the Rfam database (36, 37) predicted 44 (YeO:8 8081v) and 32 (YeO:3 Y1) riboswitch-like elements (RLEs) and RNA thermometers among the long 5′-UTRs, of which 25 were conserved in both strains (Data Sets S1-2, S1-3, S1-4, and S1-5). Several well-known RNA thermometers, e.g., the RNA thermometer of the pYV-carried regulator *lcrF* and the FMN riboswitch of *Yersiniae*, were identified (19, 38, 39), but, in addition, new interesting candidates for RNA thermometers (fimbrial mRNA *fimA-6* and the T3SS component mRNAs *yscH* and *yscD*) and riboswitches (the *crp* mRNA for the cAMP regulatory protein and the *deoC* mRNA for the deoxyribose-phosphatase synthetase) could be discovered. The presence of some elements is species or even strain specific; an RLE in the 5′-UTR of the metabolic genes *glnA*, *pepA*, and *gapA* was identified only in Y. enterocolitica and not in Y. pseudotuberculosis, and the RLE in the 5′-UTR of the peptide transporter gene *oppA* was detected only in YeO:8 8081v, whereas the RLE of the nickel/cobalt/magnesium transporter gene *corA* was found only in YeO:3 Y1 (Data Sets S1-4 and S1-5).

### The repertoire of Y. enterocolitica noncoding RNAs.

As noncoding RNAs (ncRNAs) represent an important class of posttranscriptional regulators that modulate many cellular processes, including virulence, we used the Y. enterocolitica Y1 and 8081v transcriptomes to identify noncoding RNAs (ncRNAs). Using a conservative strategy applied to Y. pseudotuberculosis (19), we were able to identify 262 (20%) and 486 (26.5%) ncRNAs in YeO:3 Y1 and YeO:8 8081v, 119 and 204 of which were expressed from intergenic regions, representing so-called *trans*-encoded small RNAs (sRNAs), and 143 and 264 from the antisense strand of mRNAs (asRNAs) (Data Sets S1-5 and S1-6). We listed them according to their location in relation to overlapping or nearby coding genes and named them Ysr(e) to distinguish them from the ncRNAs of other human-pathogenic *Yersiniae*.

A gene conservation analysis was performed by comparing the identified sRNAs in strain Y1 and 8081v with sRNAs identified in other human-pathogenic yersiniae (18, 19, 40–44). We found that only 53 sRNAs had orthologs in Y. pestis and Y. pseudotuberculosis and that only 74 of the identified Y. enterocolitica-specific sRNAs were conserved between the two strains and other members of the species (Data Sets S1-5 and S1-6). This diversity of ncRNAs was also observed among *Salmonella*, *Campylobacter*, and *Acinetobacter* species (21, 25, 29). In this context, it is assumed that ncRNAs enable rapid evolutionary fine-tuning (45), which allows the development of distinct RNA-based regulatory networks that provide the bacteria with additional species-specific or even strain-specific regulatory functions important for bacterial fitness and virulence.

**FIG 4:**
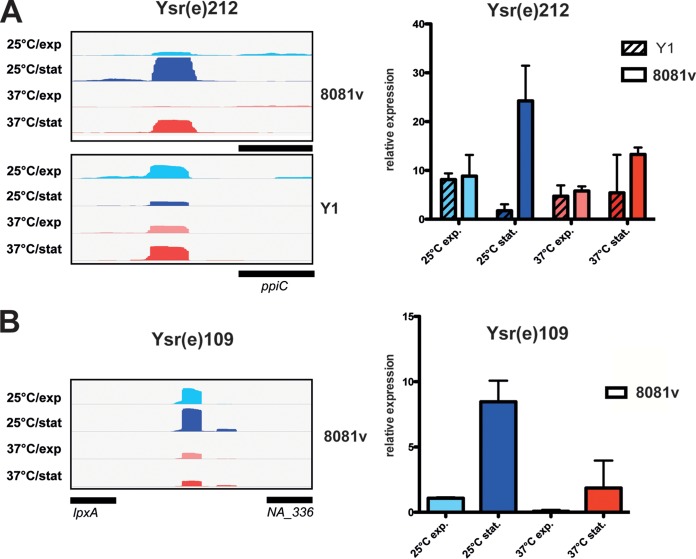
Identification of ncRNAs of YeO:3 Y1 and YeO:8 8081v. Visualization of RNA-seq-based cDNA sequencing reads of the Y. enterocolitica-specific sRNA Ysr212 (A, left panel) and the 8081v strain-specific sRNA Ysr109 (B, left panel) mapped to the 8081v and Y1 genome was performed using the IGV genome browser. Differential expression levels of the *trans*-encoded sRNAs Ysr212 of strains Y1 and 8081v (A, right panel) and Ysr109 of strain 8081v (B, right panel) determined by qRT-PCR are shown.

We validated our ncRNA identification by real-time quantitative PCR (qRT-PCR) performed with sequence-specific probes designed to hybridize to species-conserved sRNAs (e.g., Ysr021, Ysr060, Ysr143, and Ysr212), and species-specific sRNAs (e.g., Ysr109) and confirmed condition-dependent expression of the identified ncRNA transcripts (Fig. 4; see also Fig. S2). We further present a comprehensive expression landscape of all identified asRNAs in Data Sets S1-5 and S1-6. It is anticipated that the asRNAs target the complementary mRNA, whereas the biological function and the interaction partners of the *trans*-encoded ncRNAs are not easy to predict and remain to be identified in future studies.

10.1128/mSystems.00239-18.2 FIG S2 Identification of ncRNAs of YeO:3 Y1 and YeO:8 8081v. Visualization of RNA-seq-based cDNA sequencing reads of Y. enterocolitica sRNAs Ysr021 (A, left panel), Ysr060 (B, left panel), and Ysr143 (C, left panel) mapped to 8081v and Y1 was performed using the IGV genome browser. Differential expression levels of the *trans*-encoded sRNAs Ysr012 (A, right panel), Ysr060 (B, right panel), and Ysr143 (C, right panel) determined by qRT-PCR are shown. For qRT-PCR, three independent cultures of YeO:3 Y1 and YeO:8 8081v were grown in LB medium to the exponential-growth phase or the stationary-growth phase at 25°C or 37°C. qRT-PCR was performed in technical duplicate with DNA-free total RNA (primers are listed in Table S1). The *gyrB* gene was used for normalization, and relative gene expression changes were calculated according to a method previously described by Pfaffl (87). Black bars, real-time qRT-PCR for Y1; gray bars, real-time qRT-PCR for 8081v. Download FIG S2, TIF file, 1.2 MB. Copyright © 2019 Schmühl et al. 2019 Schmühl et al. This content is distributed under the terms of the Creative Commons Attribution 4.0 International license.

### Monitoring of infection-relevant changes in YeO:3 and YeO:8 gene expression.

To gain a better understanding of the genetic and molecular basis of the different host ranges and pathogenicities of YeO:3 and YeO:8 strains, we compared the levels of expression of infection-linked genes between the YeO:3 and YeO:8 strains. To do so, we first defined the core genome of both strains (3,347 genes) and profiled the entire transcriptional landscape of YeO:3 Y1 and YeO:8 8081v grown to the exponential and stationary phases at 25°C and 37°C. Comparative RNA-seq analysis was performed using DESeq2 from triplicate experiments to identify genes that are differentially regulated by at least 4-fold (*P* value of ≤0.05) in response to growth phase or temperature (Data Sets S2 and S3). The RNA-seq data visualized as circos plots show consistent expression global profiles for all conditions, with uniformly high and low transcript abundances (Fig. 1B). Despite the overall high average nucleotide identity of 97% between YeO:3 Y1 and YeO:8 8081v (Fig. S1), the bacterial expression profiles of the chromosomal genes of the two strains were distinct (Fig. 1B) and differed particularly during the stationary phase (Fig. 1C). As expected, the expression patterns of the two strains were found to be significantly different from that of Y. pseudotuberculosis YPIII (86% sequence identity; Fig. S1) under all tested conditions (Fig. 1C) (19).

**FIG 5:**
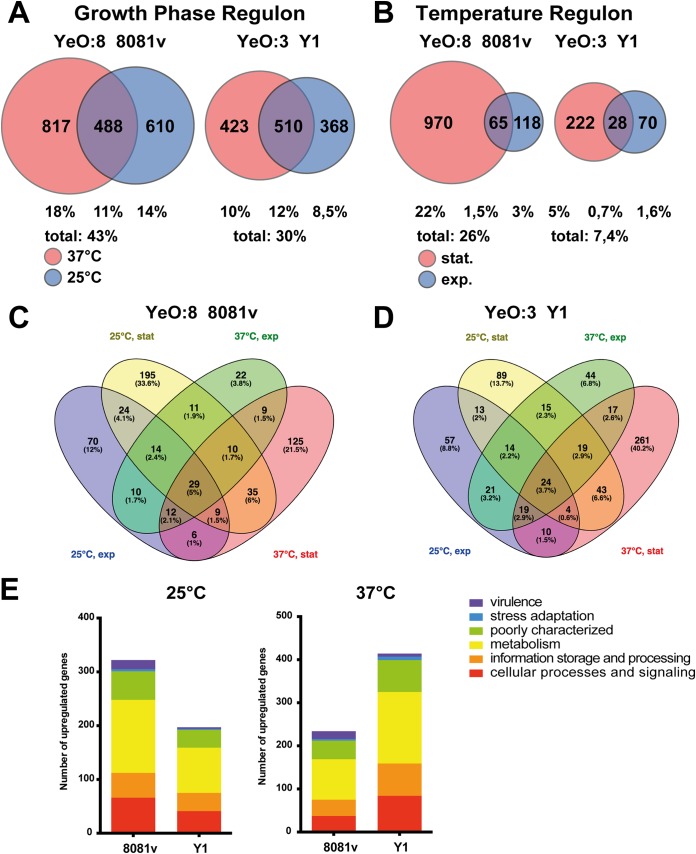
Comparison of the growth- and temperature-dependent regulons of YeO:3 Y1 and YeO:8 8081v. (A and B) Venn diagrams illustrating growth-phase-regulated (A) and temperature-regulated (B) protein-encoding genes of Y1 and 8081v. (C and D) Four-way Venn diagrams illustrating core genes that were significantly upregulated (log_2_ fold change [log_2_FC] value of ≥2; adjusted *P* value of ≤0.05) in 8081v (C) or upregulated in Y1 (D) under the indicated growth conditions. (E) Distribution of genes (categorized based on their function) that were upregulated in strain 8081v and Y1 at 25°C (left panel) or 37°C (right panel) during stationary-phase growth.

**(i) Global differences in the expression profiles of YeO:3 and YeO:8 in response to temperature.** The overall changes in the expression profiles in response to growth were comparable between the YeO:3 Y1 strain (30%) and the YeO:8 8081v strain (43%) (Fig. 5A). As expected, the levels of expression of many genes implicated in protein translation (e.g., ribosome and tRNA synthesis), cell division (*murD*, *murC*, *mraY*, *ddl*, *mrdB*/*rodA*, and *bolA*), and starvation control (*rssB*, *fadB-2*, *fadI*, and *fadH*, *psiE*) were dependent on the growth phase in both strains at both temperatures. Of the known virulence-relevant genes, several were under growth phase control only at 25°C (e.g., the catalase gene *katA* and the urease cluster *ureABCDE*) or at 37°C (e.g., fimbrial gene *fimD-1*) in both strains (Data Sets S2 and S3).

The data determined for the temperature-responsive regulons of YeO:3 Y1 and YeO:8 8081v differed more extensively than the growth-phase-control results. Observed expression changes in response to temperature were more prominent in YeO:8 8081v than in YeO:3 Y1 (Fig. 1C; see also Fig. 5B). Venn diagrams illustrate that considerably greater numbers of genes were temperature regulated (≥4-fold; *P* value of ≤0.05) in YeO:8 strain 8081v (1,153 genes [26% of the genome]) than in YeO:3 strain Y1 (320 genes [7.4% of the genome]) (Fig. 5B to D; see also Data Sets S2 and S3). This divergence is considered to be indicative of a change in the lifestyle or niche of both serotypes, in which differences in the expression patterns represent the consequences of a transition from environmental ubiquity, including in rodents and insect vectors, to specialization in enteric infection of animals such as pigs with an average body temperature of 40°C (1, 5, 7–9). In fact, among the 581 mRNAs that were more abundant in 8081v under at least one of the tested sets of conditions, about 50% were found to be more highly expressed at 37°C. This is clearly different from serotype O:3 strain Y1, in which 75% of the 650 mRNA transcripts that showed ≥4-fold-higher expression in Y1 were more abundant at 37°C (Fig. 5C to E). This set of genes includes many metabolic and cell physiology genes, indicating that the overall expression profile of the serotype O:3 strain has shifted toward higher temperatures (Fig. 5D and E; see also Data Sets S2 and S3). Global regulators (e.g., the stationary-phase sigma factor RpoS and the carbon storage regulator CsrA) and certain transcription factors that are differentially expressed in Y1 and 8081v could contribute to this process (Data Set S4).

10.1128/mSystems.00239-18.8 DATA SET S2 Global gene expression changes of Y. enterocolitica 8081 in response to temperature and growth phase. Global gene expression profiles are listed in separate sheets containing either the entire set of annotated genes (sheets S2-2, S2-4, S2-6, and S2-8) or only the genes defined as differentially expressed (sheets S2-1, S2-3, S2-5, and S2-7). Download Data Set S2, XLSX file, 2.9 MB. Copyright © 2019 Schmühl et al. 2019 Schmühl et al. This content is distributed under the terms of the Creative Commons Attribution 4.0 International license.

10.1128/mSystems.00239-18.9 DATA SET S3 Global gene expression changes of Y. enterocolitica Y1 in response to temperature and growth phase. Global gene expression profiles are listed in separate sheets containing either the entire set of annotated genes (sheets S3-2, S3-4, S3-6, and S3-8) or only the genes defined as differentially expressed (sheets S3-1, S3-3, S3-5, and S3-7). Download Data Set S3, XLSX file, 2.5 MB. Copyright © 2019 Schmühl et al. 2019 Schmühl et al. This content is distributed under the terms of the Creative Commons Attribution 4.0 International license.

10.1128/mSystems.00239-18.10 DATA SET S4 Global gene expression changes between Y. enterocolitica Y1 and Y. enterocolitica 8081v. Global gene expression changes between the two strains are listed in separate sheets containing either the entire set of annotated genes (sheets S4-1, S4-3, S4-5, and and S4-7) or only the genes defined as differentially expressed (sheets S4-2, S4-4, S4-6, and S4-8). Global gene expression changes of all core genes of Y. enterocolitica under the different tested growth conditions are listed in sheet S4-9. Download Data Set S4, XLSX file, 2.3 MB. Copyright © 2019 Schmühl et al. 2019 Schmühl et al. This content is distributed under the terms of the Creative Commons Attribution 4.0 International license.

In contrast to the chromosome results, the expression patterns of the virulence plasmid, encoding the Ysc T3SS and the antiphagocytic effectors called Yops, were comparable in the two strains. The majority of T3SS/*yop* genes are thermally induced in the two strains to similar levels, independently of the growth phase (Fig. 1C; see also Data Sets S2 and S3), suggesting that the most prominent signals triggering *ysc*/*yop* virulence gene expression are very similar in the two serotypes.

**FIG 6:**
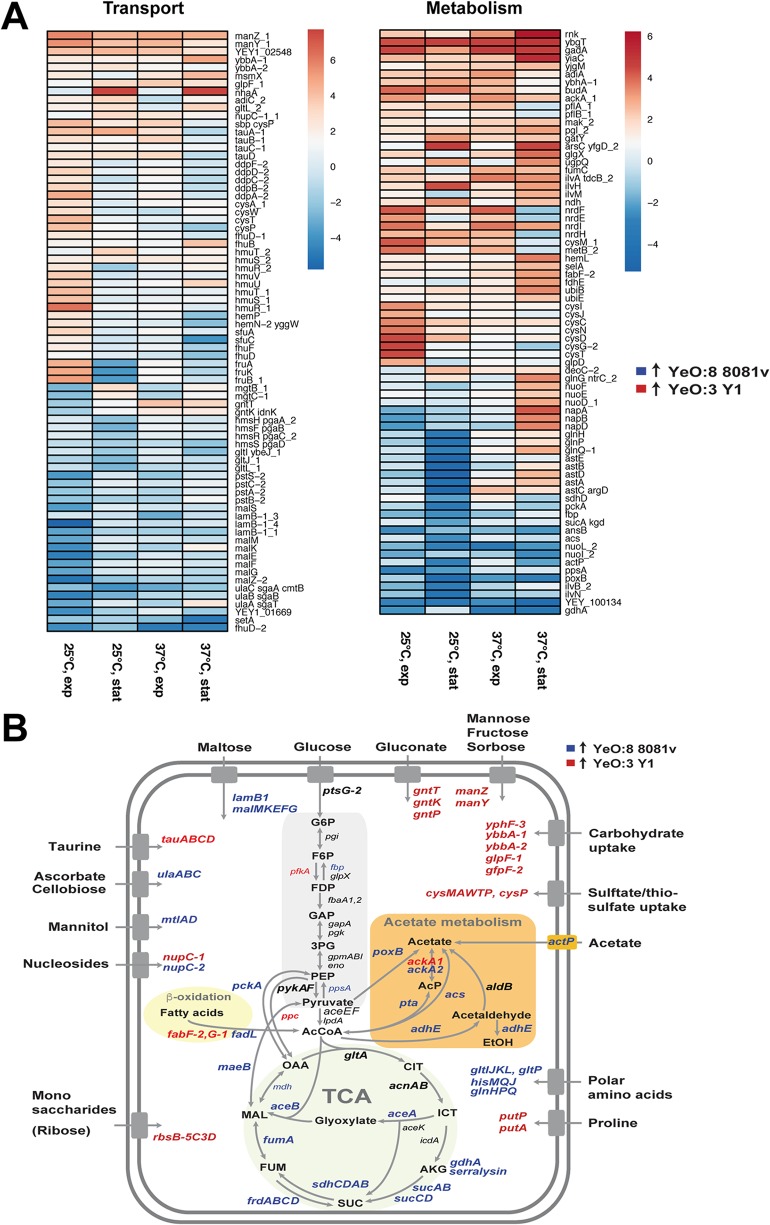
Bacterial global gene expression analysis of YeO:3 Y1 and YeO:8 8081v uncovered strain-specific metabolic and stress adaptations. (A) Heat map of selected bacterial transcripts related to metabolic and stress adaptation functions that were found to be enriched (red) or depleted (blue) in strain YeO:3 Y1 compared to YeO:8 8081v. Values represent log_2_ fold change under the indicated conditions (adjusted *P* value, ≤0.05). (B) Central carbon metabolism of Y. enterocolitica. Significant changes to the transcriptomic pattern between Y1 and 8081v grown at 25°C during the stationary phase are indicated. Enriched transcripts in Y1 are indicated in red, and enriched transcripts in 8081v are indicated in blue.

**FIG 7:**
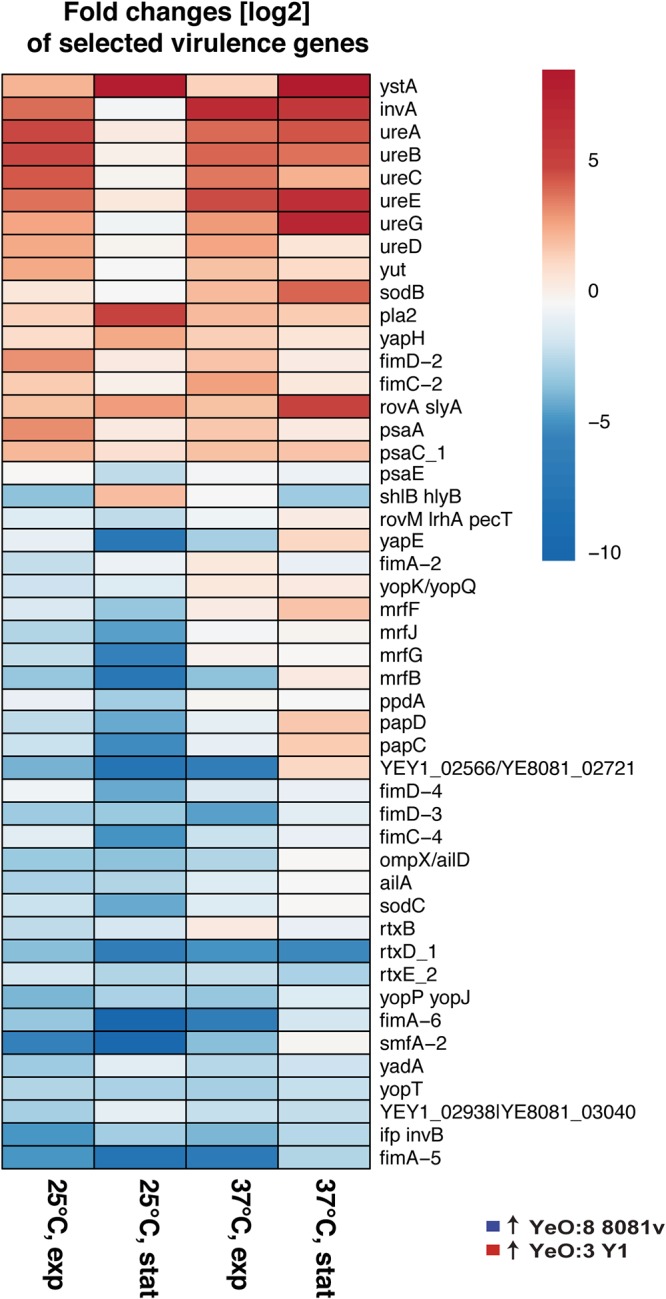
Virulence functions differentially regulated between strains YeO:3 Y1 and YeO:8 8081v. Heat maps of transcripts encoding virulence-related genes which are enriched (red) or depleted (blue) in strain YeO:3 Y1 compared to YeO:8 8081v are shown. Values represent the log_2_ fold change under the indicated conditions (adjusted *P* value, ≤0.05).

To explicitly unravel differences in the regulatory networks controlling expression of virulence-relevant genes in YeO:3 and YeO:8 strains, we used our RNA-seq approach to screen for transcripts that differed significantly in abundance between Y1 and 8081v under the different growth conditions or for genes that were coordinately regulated in response to temperature and nutrient limitation. We visualized the expression values of selected fitness- and virulence-linked genes from the two strains in heat maps (Fig. 6A; see also Fig. 7). To validate our analysis, DESeq2-estimated fold change responses for selected bacterial transcripts were confirmed by qRT-PCR (Fig. S3).

10.1128/mSystems.00239-18.3 FIG S3 Comparison of gene expression changes obtained by RNA-seq and real-time qRT-PCR. Relative gene expression changes in response to temperature or growth phase were examined for selected genes. Three independent cultures of YeO:3 Y1 and YeO:8 8081v were grown in LB medium to the exponential-growth phase or the stationary-growth phase at 25°C or 37°C. qRT-PCR was performed in technical duplicate with DNA-free total RNA (primers are listed in Table S1). The *gyrB* gene was used for normalization, and relative gene expression changes were calculated according to a method previously described by Pfaffl (87). Black bars, real-time qRT-PCR; gray bars, RNA-seq. Download FIG S3, TIF file, 1.6 MB. Copyright © 2019 Schmühl et al. 2019 Schmühl et al. This content is distributed under the terms of the Creative Commons Attribution 4.0 International license.

**(ii) Differential expression of metabolic functions indicates distinct availabilities of essential nutrients.** In total, only 24/64 mRNAs were consistently enriched (4-fold/2-fold) whereas 29/86 mRNAs were consistently depleted in YeO:3 strain Y1 compared to YeO:8 8081v under all tested conditions (Data Set S4). This set of transcripts includes several ion and nutrient transporters, e.g., the *manZY* mRNA encoding a phosphotransferase system (PTS), responsible for the transport of mannose, fructose, and sorbose family sugars, which was found to be more than 15-fold upregulated in Y1 (Fig. 5). In contrast, the ascorbate/cellobiose PTS genes (*ulaABC*) were more highly expressed in 8081v under all tested conditions. These transport functions may represent important fitness-relevant traits that allow better colonization of the bacteria in their preferred hosts.

Major differences were also observed for multiple metabolic genes; e.g., several genes encoding enzymes involved in amino acid metabolism (*gadA*, *adiA*, *ilvAHM*, *cysM*, *metB*, and *selA*) and in carbohydrate metabolism (*ackA*, *pflA*, *pflB*, *fumC*, *mak_1*, *pgl_2*, *budA*, *yiaC*, and *yjgM*) were found to be more highly expressed in Y1 whereas others, i.e., genes of the glycolysis-pyruvate-tricarboxylic acid (TCA) cycle (*gdhA*, *NA_557*/serralysin, *ansAB*, *poxB*, *ppsA*, *actP*, *acs*, *sucA*, *fbp*, and *pckA*), were more highly expressed in 8081v (Fig. 6). Overall upregulation of pyruvate-acetyl-coenzyme A (acetyl-CoA) TCA node enzymes in 8081v was particularly evident at 25°C under stationary-phase conditions, indicating that the primary carbon metabolism system has adjusted to a lifestyle associated with more frequently encountered environmental and/or insect settings, in contrast to Y1, which is a predominantly mammal-associated pathogen (Fig. 6B).

Since the number of genes that were found to be differently expressed under all tested conditions was relatively low, we hypothesized that the phenotypic differences that distinguished the serotypes were also caused by variations that modulate transcription of selected virulence genes and/or virulence-relevant fitness genes under certain growth/infection conditions. In fact, many transport and metabolic functions were found to be differentially expressed under only one of the tested conditions. Examples are the *cys* transport and metabolic genes (*cysAWTP*, *metB*, and *cysIJCNDG2M2T*), which were found to be much more highly induced in YeO:3 Y1 at 25°C during exponential-phase growth, and the *ast* operon (*astEBDA*) for amino acid metabolism, which was mainly induced in YeO:8 at 25°C during the stationary phase. In contrast, the maltose uptake system (*lamB-1*, *malS*, *malZ-2*, and *malMKEFG*) was significantly more highly expressed in YeO:8 at 25°C during exponential growth (Fig. 6A; see also Data Set S4). When the bacteria switched from the exponential phase to the stationary phase, the relative expression levels of genes involved in some metabolic functions changed. At 25°C, for instance, the fructose-specific PTS (*fruABK*) was significantly more highly induced in YeO:3 Y1 during the exponential phase but was more highly expressed in YeO:8 during the stationary phase (Fig. 6A; see also Data Set S4). The different expression patterns of metabolic functions (Fig. 6B) are likely to reflect the availability of nutrients in the intestine of the preferred hosts or insect vectors, which depends on the presence of competing microbiota and on the host diet. Moreover, distinct metabolic properties (substrate uptake and degradation) could also be advantageous during proliferation in food or environmental reservoirs.

**(iii) Comparative analysis reveals distinct mechanisms that may allow better survival in a given host.** Another major factor that determines the success of an infection in different hosts includes differences in the bacteria themselves, e.g., their ability to survive in a given host environment. A striking difference between the analyzed strains was also that some genes important for the pH resistance of bacteria were induced only in or were more highly expressed in YeO:3 strain Y1. This set includes genes of the amino acid decarboxylase/antiporter systems, depending on arginine (AdiA/AdiC) or glutamate GadC/GadA), and the urease production and urea transporter genes *ureABCEFGD* and *yut* (Fig. 6A) (Fig. 7; see also Data Set S4). These acid resistance systems bind H^+^ ions to form CO_2_ or ammonium (NH_4_^+^) (46). Moreover, *nhaA* (a strongly pH-dependent sodium/proton antiporter gene) and the *nhaR* activator gene were found to be induced more than 100-fold during the stationary phase in Y1 (Fig. 6A; see also Fig. S4B). Increased pH resistance could be advantageous for the infection of humans and larger animals (pigs/boars), in which the mean residence time in the more voluminous stomach is likely to be prolonged compared to small rodents (mice, voles, and shrews) and hares (47). On the other hand, a low level of urease activity was found to be less toxic to insects; e.g., loss of the *ureD* gene in Y. pestis was a key evolutionarily step to enable avoidance of mortality and to allow efficient transmission by fleas (48).

10.1128/mSystems.00239-18.4 FIG S4 Gene expression analysis of stress adaption genes and regulators of YeO:3 and YeO:8. Heat maps of transcripts encoding stress adaptation genes (A) or regulators (B) which are enriched (red) or depleted (blue) in strain YeO:3 Y1 compared to YeO:8 8081v are shown. Values represent log2 fold change under the indicated conditions (adjusted *P* value, ≤0.05). Download FIG S4, TIF file, 2 MB. Copyright © 2019 Schmühl et al. 2019 Schmühl et al. This content is distributed under the terms of the Creative Commons Attribution 4.0 International license.

In addition to these alterations, other fitness-relevant gene expression differences between the different serotypes were revealed. The cold shock protein genes (*cspB*, *cspC*) were generally more highly induced in YeO:8 8081v, whereas the transcripts of the universal stress genes *uspA* and *uspB*, the carbon starvation gene *cstA2*, and the *phoH* gene for a putative phosphate starvation protein were more highly expressed in YeO:3 Y1 (Fig. S4A; see also Data Set S4). Moreover, the flagellar and chemotaxis genes (*flhDC*, *motA*, *cheB-trs* [*cheD*], *fliZABCDFHIKLOR*, *flgKGCMN*, and *flhB*) were expressed only in YeO:8 8081v at 25°C during the stationary phase and not in Y1, consistent with a previous observation of our group (12). This phenotype could be linked to the expression of the autoinducer AI-2 transport and degradation system (*lsrACDBFG*), which was shown to modulate biofilm formation, chemotaxis, motility, and attachment to host cells (49). The *lsrACDBFG* operon was mainly induced at 25°C during the stationary phase in 8081v, a condition under which the level of expression of the *lsrRK* mRNA, encoding the equivalent repressor LsrR and the regulator LsrK, was more than 4-fold lower (Fig. S4A).

**(iv) Differential expression of colonization, dissemination, and toxin genes.** Several genes have been identified that are important for *Yersinia* virulence, host colonization, and antimicrobial resistance. Our data set offers a detailed and comparative molecular analysis of the transcript abundances of well-characterized and less extensively characterized Y. enterocolitica virulence factors. Fimbrial and nonfimbrial adhesins are critical components in Y. enterocolitica virulence, as they are required for efficient host cell binding to colonize the intestinal tract and subepithelial tissue. Several adhesin genes are differentially regulated between YeO:3 Y1 and YeO:8 8081v. Prominent examples are the fimbrial clusters *mrfJHGF-papCD-mrfB-smfA2*, *fimA5-NA_360-fimD3-NA_361-NA_362*, and *fimA6-fimC4-fimD-4-NA_538*/*6-NA_539*/*5*, which were shown to be more highly expressed in YeO:8 8081v, whereas the PsaA antigen/fimbria genes and the *fimD2-fimC2* genes were more highly induced in YeO:3 Y1 (Fig. 7; see also Data Set S4). In addition, transcripts for the afimbrial adhesins, such as the outer membrane adhesin YapH and the primary cell adhesion and invasion factor InvA, were significantly more abundant in Y1 (Fig. 7; see also Data Set S4). The latter observation is consistent with the results from our previous study demonstrating an increased synthesis of InvA in porcine and human isolates of YeO:3 strains due to the presence of a constitutive promoter of an IS*1667* element integrated into the *invA* promoter region and to overall higher expression levels of the transcriptional activator RovA of *invA* (12) (Fig. 7; see also Data Set S4). In contrast, larger amounts of the mRNAs of the attachment and invasion locus AilA, the homologous protein AilD/OmpX, the InvA-type adhesin InvB/Ifp, and the virulence plasmid-encoded adhesin YadA were detected in 8081v. This likely mirrors the selectivity/preference of the serotype O:3 and O:8 strains for certain host cells expressing the adhesin-specific cellular receptor. Alteration of the pathogen-host cell interactions modulates the colonization and/or dissemination behavior of the bacteria and may promote a serotype-specific preference for different hosts, e.g., humans, pigs/boars, hares, or small rodents. Many of these colonization factors were strongly controlled in response to temperature and growth phase in YeO:8 strain 8081v but were more equally expressed in YeO:3 strain Y1. The most prominent difference, in which the fimbrial cluster mRNA (*mrfJHGF-papCD-mrfB-smfA2*, *fimA5-NA_360-fimD3-NA_361*, and *fimA6-fimC4-fimD-4-NA_538*/*6-NA_539*/*5*) was significantly more abundant in YeO:8 strain 8081v, was observed at 25°C during the stationary phase (Fig. 7; see also Data Set S4). Expression of these adhesion structures at moderate temperatures may be important to colonize certain environmental reservoirs and insect vectors and/or to prime the bacteria to allow immediate and efficient colonization of the intestinal epithelium upon host entry.

Another major factor that affects host specificity includes differences in the ability of the bacteria to evade a given host immune response. Notably, two effector proteins, YopT and YopJ/YopP, which were injected into neutrophils and macrophages to perturb host innate immune responses, were more highly expressed in YeO:8 strain 8081v (Fig. 7; see also Data Set S4). YopT, a cysteine protease targeting the small GTPases Rac1, RhoA, Cdc42, and RhoG, is implicated in the disruption of the actin cytoskeleton to contribute to the inhibition of phagocytosis. YopP/YopJ interferes with multiple IκB kinase β (IKKβ)-signaling/NF-κB-signaling and mitogen-activated protein kinase (MAPK)-signaling components to inhibit proinflammatory cytokine and chemokine production and activates caspase-1 and the maturation of IL-18 and IL-1β to induce immune cell death (50). This suggests that a higher level of activity with respect to inhibiting and eliminating host phagocytes is required or advantageous for 8081v to survive in its preferred hosts. In contrast, the outer membrane protease Pla-2 and one of the most important virulence factors of Y. enterocolitica, the heat-stable enterotoxin A (YstA), were shown to be significantly more highly expressed in YeO:3 strain Y1 (Fig. 7; see also Data Set S4), suggesting significantly higher toxicity of this strain.

### Differential expression of the *ystA* toxin gene.

The YstA enterotoxin is one of the most important and reliable virulence markers of Y. enterocolitica. It strongly influences *Yersinia* virulence and is a major causative agent of secretory diarrhea. In one study, all 89 of 89 pathogenic and none of 51 nonpathogenic Y. enterocolitica isolates contained *ystA*-homologous genes (51). Moreover, rabbits infected with a *ystA*-positive (*ystA*^+^) strain suffered from diarrhea and rapidly lost weight, and most died, whereas rabbits infected with the *ystA* mutant showed no disease symptoms, and the strain rapidly disappeared from the feces (52). The mechanism of YstA action is based on guanylate cyclase activation, which results in increased levels of cGMP in enterocytes and of extracellular liquids in the intestines (53, 54). It further increases the levels of intracellular inositol triphosphate (IP3), interacting with the IP3 receptor and mobilizing intracellular calcium in intestinal epithelial cells (55).

**FIG 8:**
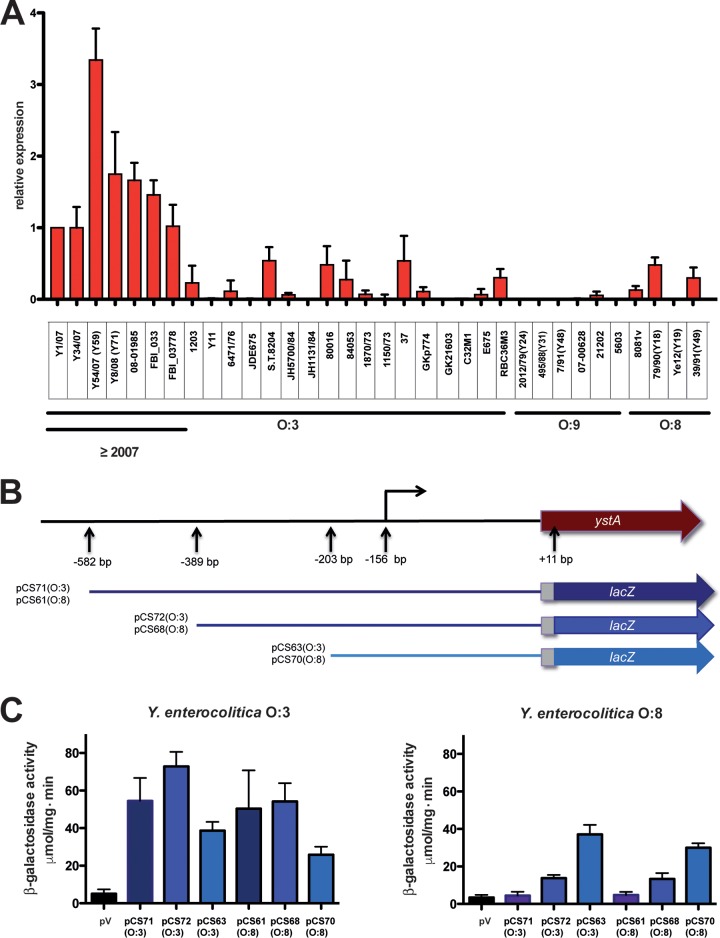
Analysis of *ystA* expression in Y. enterocolitica. (A) RNA was isolated from three independent cultures of different Y. enterocolitica serotype O:3, O:8, and O:9 isolates grown to the stationary-growth phase at 25°C. Expression of *ystA* relative to that seen with Y. enterocolitica Y1 was determined using qRT-PCR. qRT-PCR was performed in technical duplicate with DNA-free total RNA (primers are listed in Table S1). The 5S rRNA gene was used for normalization, and relative gene expression changes were calculated according to a method previously described by Pfaffl (87). The data represent the means ± standard errors of the means (SEM) of the relative expression levels determined in three independent biological replicates performed in triplicate. (B) The scheme illustrates the constructed plasmid-carried *ystA-lacZ* translational fusion harboring different portions of the *ystA* promoter region. The numbers (i) indicate the positions relative to the TSS used for the cloning of the fusions or (ii) indicate the identified promoter. (C) The *ystA-lacZ* translational fusions harboring the 5′-UTR of the *ystA* gene of Y1 or 8081v were transformed either in Y1 or into 8081v. The levels of β-galactosidase activity were determined. The data represent means ± SEM of the fold change (end/start) values from three independent biological replicates performed in triplicate and were analyzed with Student's *t* test.

Our comparative RNA-seq analysis revealed that the gene of the YstA toxin (*ystA*) was significantly more highly expressed in serotype O:3 strain Y1 than in serotype O:8 strain 8081v (Fig. 7; see also Data Set S4). Next, we compared the abundances of the *ystA* transcript at 37°C during the stationary phase for Y1 and a group of *ystA*-positive clinical isolates of Y. enterocolitica representing different biotypes isolated from distinct geographical regions of the world at different time points. All isolates of serotypes O:8 and O:9, as well as “older” isolates of YeO:3 (collected before 2007), exhibited differing but, in general, very similar low expression levels of the toxin (Fig. 8A). In contrast, most of the isolates obtained over the past 10 years produced higher levels of the *ystA* transcript (Fig. 8A). It is possible that the older isolates switched *ystA* expression to a silent state such as has been described previously for some isolates (56). Alternatively, the more recent strains might have acquired an additional mutation leading to an increase of *ystA* gene transcription or *ystA* mRNA stability. As the *ystA* promoter region of the YeO:3 strains Y11 and 1203 with low *ystA* transcript levels (Fig. S5) is 100% identical to that of YeO:3 Y1 with high *ystA* mRNA amounts, we assumed that the distinct expression levels seen represent the results of differences in a *trans*-encoded factor. In fact, transcriptional *ystA-lacZ* fusions harboring the entire *ystA* promoter region of Y1 and 8081v (positions −582 to +11 with respect to the translational start site) were both highly expressed in YeO:3 strain Y1 and fully repressed in YeO:8 strain 8081v (Fig. 8B and C).

10.1128/mSystems.00239-18.5 FIG S5 Promoter region of the *ystA* gene in different Y. enterocolitica strains. A sequence comparison of the *ystA* promoter region of YeO:3 strains Y1, Y11, and 1203 and of YeO:8 strain 8081v is shown. The coding region is marked with a blue box and the AT-rich regions with a red box. The identified transcriptional start site is indicated by a broken arrow, and the putative −10 and −35 regions are marked with a bar. Download FIG S5, TIF file, 1.2 MB. Copyright © 2019 Schmühl et al. 2019 Schmühl et al. This content is distributed under the terms of the Creative Commons Attribution 4.0 International license.

10.1128/mSystems.00239-18.6 TABLE S1 Bacterial strains, plasmids, and oligonucleotides. Download Table S1, DOCX file, 0.03 MB. Copyright © 2019 Schmühl et al. 2019 Schmühl et al. This content is distributed under the terms of the Creative Commons Attribution 4.0 International license.

**FIG 9:**
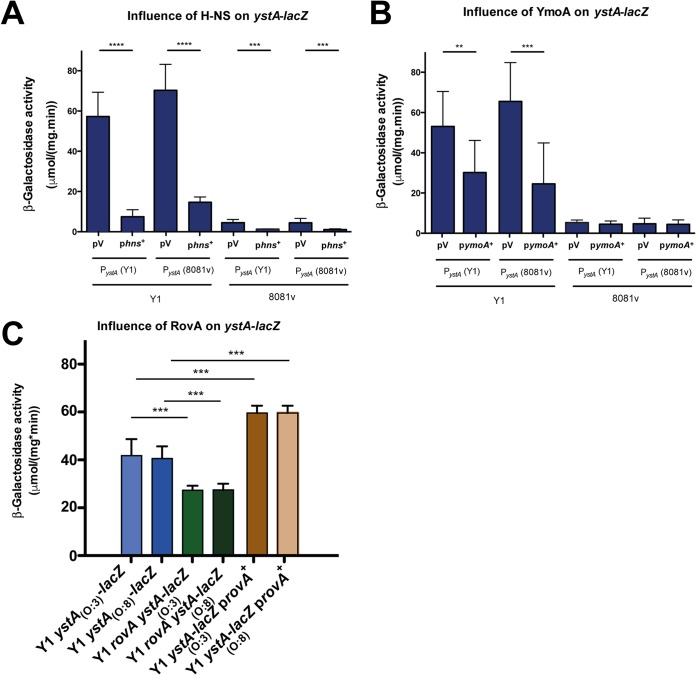
Influence of H-NS, YmoA, and RovA on *ystA* expression of Y. enterocolitica strain Y1. Plasmids carrying the *hns* gene (A), the *ymoA* gene (B), or the *rovA* gene (C) were transformed into YeO:3 strain Y1 or the isogenic *rovA* mutants carrying a *ystA-lacZ* fusion construct with the entire *ystA* promoter region of the *ystA* gene of Y1 or 8081v. The strains were grown to the stationary phase at 25°C, and β-galactosidase activity was determined. The data represent means ± SEM of the fold change (end/start) values from three independent biological replicates performed in triplicate and were analyzed with Student's *t* test. The stars indicate the results that differed significantly from those of the wild type harboring the identical reporter plasmid as follows: **, *P* < 0.01; ***, *P* < 0.001; ****, *P* < 0.0001.

Different deletions of the *ystA* promoter region resulted in progressive increases in the level of *ystA* transcription in YeO:8 8081v (Fig. 8B and C), suggesting that an additional negative regulatory protein represses *ystA* expression but that this silencing is relieved in YeO:3 Y1. Close inspection of the *ystA* upstream region revealed high levels of AT abundance and the occurrence of long poly(AT)-rich stretches upstream of the transcriptional start site overlapping the identified promoter in this study (Fig. S5) and in a previous study (56). This indicates a high level of DNA flexibility and characterizes the predominant binding and nucleation sites of the global nucleoid-associated regulator H-NS (57, 58). Interaction of H-NS with these sites leads to polymerization and the formation of higher-order nucleoprotein complexes, resulting in the repression of the target promoter downstream (59). To investigate a potential role of H-NS in silencing of *ystA* in 8081v, which seems to have been eliminated in Y1, we measured the levels of expression of the *ystA-lacZ* fusion in Y1 in the presence of a *hns*^+^ plasmid and found that epitopic expression of the *hns* gene led to a strong repression of *ystA* in a manner very similar to what is seen in 8081v (Fig. 9A). A similar influence was observed for YmoA (Fig. 9B), an H-NS homologue, which interacts directly with H-NS and forms a repression complex silencing a subset of H-NS-controlled virulence genes (60). This strongly indicated that H-NS/YmoA-mediated repression of *ystA* in 8081v is relieved in Y1, potentially by an activator protein that counteracts the function of H-NS. One obvious candidate for that activator protein is RovA. RovA was shown to counteract H-NS and YmoA-regulated genes in *Yersinia*, including *invA* and *psaA* (60–62), which were both upregulated in Y1 compared to 8081v (Fig. 7; see also Data Set S4). In fact, *rovA* expression was found to be much more highly induced in YeO:3 Y1 than in YeO:8 under all tested conditions but in particular during the stationary phase, in which the expression level of the *ystA* transcript was mostly increased (Fig. 7; see also Data Set S4). This is in full agreement with results from a previous study by our group showing that the amount of RovA in YeO:8 8081v (and in YeO:3 strain Y11, with low *ystA* transcript levels) was lower than that in YeO:3 Y1 (12). This was caused by a P98S substitution in RovA. This amino acid exchange renders the regulator less susceptible to proteolysis and results in a more efficient autoactivation of its transcription (12). We tested whether a mutation in *rovA* and overexpression of *rovA* would influence expression of the *ystA*_O:3_*-lacZ* and *ystA*_O:8_*-lacZ* fusions and found that RovA was able to enhance *ystA* expression in Y1 (Fig. 9C). A report from a previous study also indicated that YstA requires the ncRNA chaperone Hfq for maximal expression (63). That observation can now be explained by the requirement of Hfq for the stability of the regulatory RNAs CsrB and CsrC, which positively influence expression of YstA via the CsrA-RovM-RovA cascade (64).

### Conclusions.

The ability of pathogenic bacteria to reprogram their fitness- and virulence-related traits can enable them to adapt to other environmental reservoirs and hosts. This can lead to unexpected outbreaks and epidemics in distinct host species populations and thus represents a global public and veterinary health concern. To obtain information about the molecular basis of host tropism, population genomic studies have primarily been applied. Those studies have provided data on the core genome of the genus and led to the identification of specific point mutations (single nucleotide polymorphisms [SNPs]) and of gene gain, gene loss, and genome rearrangement events that influence host adaptation pathways and specificity in *Yersinia* and other bacterial pathogens (5, 13, 65–68). Among the functions that were altered as different Y. enterocolitica lineages evolved and adapted to new host niches was the cell adhesion and invasion factor InvA. In the highly mouse-virulent phylogroup 2/serotype O:8 strains, *invA* is strongly temperature regulated and is predominantly transcribed at 25°C during the stationary phase. However, in phylogroup 3/serotype O:3 strains, which show limited pathogenesis in mice but have become the dominant isolate found in pig reservoirs and cases of human disease, an IS*1667* element was found to have integrated into the *invA* promoter. This created a new promoter and an additional binding site for the RovA activator that ensures constitutive expression of the invasin gene (12, 69). The upregulation of *invA* enabled a more efficient colonization of porcine tissue than was seen with other phylogroups (16), suggesting that this is the primary event that led to the enhanced virulence observed in recent isolates from the phylogroup 3/O:3 strains.

In this study, we followed a different approach and compared the first primary transcriptomes of Y. enterocolitica by the use of strains 8081v and Y1, representing phylogroups 2 and 3, to determine the transcriptional variability in the response to infection-relevant conditions. This revealed strain-specific promoter usage and sRNA repertoires and uncovered different transcriptional outputs that are also likely to facilitate adaptation to different host niches and impact pathogenesis. Integration of the comparative double-stranded RNA (dRNA)-seq data from the two strains under four different growth conditions improved the annotation accuracy and allowed us to determine 1,299 and 1,076 TSSs of mRNAs in 8081v and Y1, respectively, the majority (1,213 and 1,043, respectively) of which belong to the core genome and are conserved between the two strains. However, many examples of strain-specific promoter usage were identified also, and although some promoters were found to be highly conserved, the respective genes are not necessarily expressed at the same level by Y1 and 8081v. One prominent example is the *ystA* gene, which is strongly induced in Y1, in particular, during the stationary phase, but not in 8081 and other older serotype O:3 isolates with an identical promoter region. This illustrates that comparative transcriptomics is an excellent approach to discover differences in the functional output from genomes which cannot be directly inferred from examination of closely related DNA sequences.

Overall, our high-resolution transcriptome map discovered major differences between the phylogroup 2 and 3 strains in the transcription patterns, in particular, differences in transcription of the temperature-responsive regulon. Multiple fitness- and virulence-relevant genes were found to be controlled in response to temperature and were often expressed at a higher level at 25°C in the serotype O:8 strain 8081v, whereas no significant thermal response or a much less extensive thermal response was observed in the homologous genes in serotype O:3 isolate Y1. This most likely reflects differences in the lifestyles of the bacteria and points to a recent study proposing ecological separation from certain niche-adapted pathogenic lineages of Y. enterocolitica (6). Although all phylogroups of Y. enterocolitica can be isolated from the intestinal tract of cattle sheep and pigs, serotype O:8/phylogroup 2 strains have rarely been isolated from humans and livestock and have been shown to have a higher level of virulence in mouse infection models. Moreover, results of analyses of the core and accessory genes and of the gene flow across the phylogroups suggest that the different phylogroups are ecologically separated and do not share common niches (6, 70).

Observed genetic and transcriptional differences can be adaptive and lead to niche expansion/separation. A variety of pathoadaptive alterations were identified which can affect (i) host cell binding, colonization dissemination, and host tissue tropism; (ii) the pathogen’s ability to evade or overcome immune mechanisms; (iii) the ability of the pathogen to survive stresses; (iv) uptake and utilization of essential nutrients for growth; and (v) virulence regulation. All these features are important for virulence and determine host specificity/tropism (50). The most striking differences have been determined for the acid resistance genes, the adhesins, and the YstA enterotoxin. *ystA* mRNA is much more abundant in Y1 than in 8081v. This indicates a much higher level of toxicity of Y1. However, exotoxin function is linked to the ability of the pathogen to adhere to the intestinal epithelial layer; i.e., the bacteria require a colonization factor that promotes tight interaction with intestinal epithelial cells for the onset of diarrhea. Some Y. enterocolitica fimbriae and the afimbrial adhesin invasin (InvA), which are more strongly expressed in YeO:3 Y1 at body temperature, are likely candidates, as they guarantee that the serotype O:3 strains are much better colonizers of the pig intestine than the serotype O:8 strains (14, 16). Enhanced expression of the YstA toxin marked strong diarrhea of the patients from whom these isolates were cultured. How this combination of adhesion factors and the toxin impacts pathogenesis needs to be characterized in future studies. However, it is notable that a similar cocktail of virulence factors leading to a more efficient form of aggregative adherence by newly emergent Escherichia coli serotype O104:H4 was shown to account for the increased uptake of Shiga toxin into the systemic circulation, resulting in high rates of the hemolytic-uremic syndrome (71).

## MATERIALS AND METHODS

### Bacterial strains.

All Y. enterocolitica strains were grown in Luria broth (LB) to exponential phase (optical density at 600 nm [OD_600_] of 0.5) or stationary phase (16 h) at 25°C and 37°C under anaerobic conditions for RNA isolation and RNA-seq analysis. Bacteria were cultivated in brain heart infusion (BHI) medium for transformation with the indicated plasmids. E. coli was grown at 37°C in LB medium. If necessary, antibiotics were added at the following concentrations: kanamycin 50 µg ml^−1^, chloramphenicol 30 µg ml^−1^. All strains used in this study are listed in Table S1 in the supplemental material.

### DNA manipulation and plasmid construction.

PCR amplification, restriction digestions, ligations, and transformations were performed using standard genetic and molecular techniques (72, 73). The plasmids used in this work are listed in Table S1. Oligonucleotides used for PCR and qRT-PCR were purchased from Metabion and are listed in Table S1. Plasmid DNA was isolated using a Nucleospin plasmid kit (Macherey & Nagel, Germany). Restriction enzymes and DNA-modifying enzymes were purchased from New England Biolabs. PCRs were performed in a 50-µl volume for 29 cycles using Phusion High-Fidelity DNA polymerase (New England Biolabs) or *Taq* polymerase (Promega). Purification of PCR products was performed using a Nucleospin Gel and PCR Clean-up kit (Macherey & Nagel, Germany). The resulting plasmids were sequenced by Seqlab (Göttingen, Germany). Plasmids pCS71, pCS72, and pCS63 were constructed by amplifying the 5′-UTR of *ystA* from genomic DNA of YeO:3 Y1 with forward primers VIII009, VIII010, and VIII011 and reverse primer VIII016. Plasmids pCS61, pCS68, and pCS70 were constructed by amplifying the 5′-UTR of *ystA* from genomic DNA of YeO:8 8081v with forward primers VIII009, VIII014, and VIII015 and reverse primer VIII019. The PCR-derived fragments were integrated into the XhoI/NheI site of pFU55 (74), creating fusions of the 5′-UTR to *lacZ*. Plasmid pHT109 was constructed by amplifying the *rovA* gene (with its own promoter) using primers 123 and 508. The fragment was inserted into pZA31 using the KpnI and ClaI restriction sites.

### YeO:3 strain Y1 genome sequencing and annotation.

Y. enterocolitica strain Y1 deposited at the DSMZ (Deutsche Sammlung von Mikroorganismen und Zellkulturen) (no. 107832; NCBI accession no. CP030980 and CP030981), representing a recently collected O:3/4 human isolate (12), was selected to be the reference strain and sequenced. Genomic DNA of Y1 was isolated using a Qiagen genomic-tip 100/G kit (Qiagen, Germany). DNA concentrations were measured using a Qubit fluorometric quantitation system (Thermo Fisher Scientific, USA) and adequate quality was verified using pulsed-field gel electrophoresis. The genomic sequence was determined using PacBio *RSII* and an Illumina Hiseq 2500 system.

A SMRTbell template library was prepared according to instructions from Pacific Biosciences, Menlo Park, CA, USA, following the “Procedure & Checklist—Greater Than 10 kb” template preparation method. Briefly, for preparation of 15-kb libraries, 8 µg genomic DNA was sheared using g-tubes from Covaris, Woburn, MA, USA, according to the manufacturer´s instructions. DNA was end repaired and ligated overnight to hairpin adapters, applying components from DNA/Polymerase Binding kit P6 from Pacific Biosciences, Menlo Park, CA, USA. Reactions were carried out according to the manufacturer´s instructions. BluePippin selection of sizes to greater than 4 kb was performed according to the instructions of the manufacturer (Sage Science, Beverly, MA, USA). Conditions for annealing of sequencing primers and binding of polymerase to a purified SMRTbell template were assessed with the Calculator in RS Remote (Pacific Biosciences, Menlo Park, CA, USA). Single Molecule, Real-Time (SMRT) sequencing was carried out on a PacBio *RSII* system (Pacific Biosciences, Menlo Park, CA, USA), recording the results in one 240-min movie.

The PacBio run yielded 70,767 reads with a mean read length of 12,720 bp. SMRT cell data were assembled using the “RS_HGAP_Assembly.3” protocol included in SMRT Portal version 2.3.0 and default parameters. The assembly revealed a circular chromosome (YEY1_1) and one circular plasmid (YEY1_2). Both replicons were circularized; in particular, artificial redundancies at the ends of the contigs were removed and adjusted to *dnaA* and *sopB* as the first genes. Error correction was performed by a mapping of 1.4 million paired-end reads of 2 × 301 bp generated on an Illumina MiSeq system onto finished genomes using Burrows-Wheeler alignment (BWA) (75) with subsequent variant and consensus calling using VarScan (76). A consensus concordance quality value of 60 (QV60) was confirmed for the genome. Finally, an annotation was carried out using Prokka 1.8 (77). Thereby, an optional user-provided set of annotated proteins was used as the primary source of annotation, containing the annotation information of all genes in Y. pseudotuberculosis YPIII. The average GC content was 47%, a value similar to that determined for Y. enterocolitica strain 8081 (NC_008800; 47.27%). The complete Y1 genome sequence was deposited in NCBI under accession numbers CP030980 (chromosome YEY1_1) and CP030981 (plasmid YEY1_2).

### RNA isolation.

Y. enterocolitica 8081v and Y1 were grown in LB medium to the exponential phase (OD_600_ of 0.5) and the stationary phase (16 h) at 25°C and 37°C, respectively. Total bacterial RNA was isolated by the use of a hot phenol extraction protocol (73). The remaining DNA was digested using Turbo DNase (Ambion), and RNA was purified with phenol:chlorophorm:isoamylalcohol. The quality was assessed using an Agilent RNA 6000 Nano kit on an Agilent 2100 Bioanalyzer (Agilent Technologies). From 5 µg of total RNA, the rRNA was depleted using RiboZero (Illumina).

### Strand-specific library preparation and Illumina sequencing.

Strand-specific RNA-seq cDNA library preparation and barcode introduction were performed using a NEBNext multiplex small RNA library preparation set for Illumina (New England Biolabs). In brief, the rRNA-depleted RNA was fragmented by sonication to a median size of 200 nt. The fragments were 5′ phosphorylated and ligated to 3′- and 5′-RNA-adapter oligonucleotides. After reverse transcription, cDNA libraries were subjected to PCR amplification (15 cycles). The quality of the libraries was validated using an Agilent 2100 Bioanalyzer (Agilent Technologies) following the manufacturer’s instructions. Single-end sequencing on a HiSeq 2500 system was performed with 2 nM library concentrations denatured with 0.1 N NaOH and diluted to a final concentration of 12 pM. Cluster generation on HiSeqSR Flow Cell v3 was generated at cBot using TruSeq SR cluster kit v3-HS to create single-molecule DNA templates followed by bridge amplification. Sequencing runs were performed using a HiSeq 2500 system and TruSeq SBS kit v3 (50 cycles) to run 51 cycles and 7 cycles for the single-indexed read. The fluorescent images were processed to sequences and transformed to FastQ format using Genome Analyzer Pipeline Analysis software 1.8.2 (Illumina). The sequence output was controlled for general quality features, sequencing adapter clipping, and demultiplexing using the fastq-mcf and fastq-multx tool of ea-utils: Command-line tools for processing biological data (78).

### Read mapping, bioinformatics, and statistics.

The quality of the sequencing output was analyzed using FastQC (Babraham Bioinformatics). All sequenced libraries were mapped to the YeO:8 8081v genome (NC_008800.1) and pYVO:8 plasmid (NC_008799.1) or the YeO:3 Y1 genome (CP030980) and pYVO:3 plasmid (CP030981) using fast-gapped read alignment tool Bowtie2 (79) with default parameters. After read mapping, SAMtools (80) was employed to filter the resulting bam files for uniquely mapped reads (on both strands). Reads were classified as uniquely mapped with a unique genomic location if and only if they could not be aligned to another location with higher or same mapping quality. The resulting bam files constituted the basis for all downstream analyses and were used for visualization. (For detailed mapping statistics, see Data Set S1 in the supplemental material.) Obtained data were further processed as described previously (18, 19).

### Detection of transcriptional start sites.

To detect transcriptional start sites, libraries treated with 5′ polyphosphatase (+Phos) were compared to libraries not treated with 5′ polyphosphatase (−Phos), which provides the background distribution of read starts. The −Phos libraries are depleted for cDNA derived from fragments containing the 5′ end of primary transcripts, while the corresponding +Phos libraries are unbiased. To verify the transcriptional start sites, additional libraries treated with TEX (terminator exonuclease) were compared to the −TEX libraries. The TEX-treated libraries are enriched for primary transcripts, as TEX digests RNAs with 5′ monophosphate but not 5′ triphosphate. In the first step, sample libraries were normalized to 1 million uniquely mapped reads and the coverage and the numbers of reads starting at the respective positions were calculated for every base. Then, biological replicates were combined/merged by averaging the coverage and read start data. To detect the transcriptional start sites for YeO:8 8081v and YeO:3 Y1, we applied TSSAR (81) to the RNA-seq data. All TSSs obtained from TSSAR were inspected manually and curated. In cases in which a sharp 5′ flank cDNA read (≥10 reads) with continuous coverage with respect to a downstream gene was manually detected, this position was added to the set of mRNA TSSs, although TSSAR failed to detect the respective TSS.

TSSs were assigned to four different categories (82). If a TSS was located upstream of an annotated gene, it was assigned the designation “mTSS” (TSS of mRNA). When a TSS matched the position of the translation start codon but was within 10 nt of the translational start codon, the TSS was assigned the designation “lmTSS” (TSS of leaderless transcript). TSSs of *cis*-encoded antisense RNAs that were oriented antisense to a protein-coding gene with no continuous coverage to the gene located downstream were assigned the designation “asTSSs” (antisense RNAs). If a TSS was located in an intergenic region with an appropriate distance from and no coverage with respect to the next start codon, it was assigned the designation “sRNA” (*trans*-encoded RNA). Adjacent TSSs (separated by less than 3 nt) were clustered, and the TSS with the highest number of read start counts was annotated as the TSS corresponding to that cluster. The newly identified TSSs were labeled according to the following convention: x_TSS_CDS_n, where “x” indicates strain Y1 or 8081v. TSSs that were assigned to protein-coding genes were compared between YeO:8 8081v and YeO:3 Y1. TSSs were considered to be conserved between the two strains if they were assigned to the same gene and located at the same distance (±5 nt) from the translational start codon.

### Detection of conserved sequence motifs.

To investigate potential sequence conservation at the determined TSS, a sequence logo for position +1 to position +3 (with the TSS representing position +1) of all 1,299 TSSs for 8081v and 1,076 TSSs for Y1 was generated using WebLogo software (83). We performed *de novo* motif discovery using MEME software (24) to compute conserved sequence motifs in the −10 and −35 promoter regions. Subsequences starting at position −15 and ending at position −3 (relative to the TSS) of all TSSs determined for each strain served as the input for motif detection in the −10 region. For the −35 region, we used subsequences starting at position −45 and ending at position −25. We ran MEME in zero or one occurence per sequence (ZOOPS) mode and searched for motifs between length 3 and length 8 for the −10 region and between length 3 and length 5 for the −35 region.

### Identification of small regulatory RNAs (sRNAs).

To identify expressed sRNAs, a global screen in all samples for unannotated *trans*-encoded sRNAs and *cis*-encoded antisense RNAs was performed as described previously (19). In brief, transcripts were assembled from reads and classified. For sRNA classification, TSS data were included in the annotation of Y. enterocolitica strain Y1 and 8081v. In the first step, transcripts seeds, which corresponded to genomic regions of a minimal length of 40 nt and continuous coverage of at least 30 reads were considered candidates for sRNAs. The resultant transcripts were extended on both ends until the level of coverage was lower than 3 reads. Finally, transcripts located in intergenic regions without overlapping UTRs were classified as *trans*-encoded sRNAs, while transcripts found on the strand opposite to a protein-coding gene were defined as *cis*-encoded antisense RNAs. All sRNA candidates were inspected manually and checked if they passed that last filter. The novel noncoding RNAs were labeled according to the common convention [Ysr(e)_n] with ongoing numbers (n). Identified sRNA candidates were compared between YeO:8 8081v and YeO:3 Y1 based on BLASTN analysis and the genomic context. Levels of conservation of sRNAs within other *Yersinia* species and gammaproteobacteria were determined by BLASTN analysis. RNA sequences were used to scan Rfam (36, 37) for related sequences.

### Differential expression analysis.

Reads aligned to annotated genes were quantified with the htseq-count program (84). To detect genes that were differentially expressed in 8081v and Y1, we employed DESeq2 (version 1.2.1) (85). For DESeq2 parameterization, we used a beta prior and disabled the Cook distance cutoff filtering. All other parameters remained unchanged. HTSeq in union count mode was used to generate the raw read counts required by DESeq2 as the basis for differential expression analysis. In addition, RPKM (reads per kilobase per million per mapped reads) values were computed for each library from the raw gene counts. The list of DESeq2-determined differentially expressed genes (DEGs) was filtered with a conservative absolute log_2_ fold change cutoff value of at least 2 and a cutoff value for a multiple-testing-corrected *P* value of at most 0.05.

To assess platform dynamic range and the accuracy of fold change response, we used ERCC RNA spike-in controls (Thermo Fisher Scientific). Spike-in control sequences were added to the reference genome/annotation prior to read alignment, and read counts for spike-in controls were determined along with normal gene counts using the htseq-count program.

### Cross-species analysis.

To allow comparison of the transcriptomes of Y. enterocolitica strain Y1 and strain 8081v and construction of a correspondency table of locus tags, we computed a bijective mapping of all coding genes by reciprocal-best BLASTP (86) hits with an E value cutoff of 1.0E−6. By using this mapping table, we were able to construct raw read count matrices containing corresponding counts from both of the species and to use them for cross-species DEG analysis with DESeq2. To construct the core proteome and to compare the expression profiles of more than two *Yersinia* transcriptomes (see Fig. 1C), we clustered all protein coding genes on the basis of the results from an all-versus-all BLAST comparison. More precisely, we computed the core proteome of the number (*N*) *Yersinia* strains by finding cliques of size *N* in the graph of reciprocal best BLASTP hits across species boundaries, where each clique contained exactly one member of each of the *N* involved strains. The set of identified cliques allowed us to construct a correspondency table for core genes of more than two strains reflecting orthologous gene relationships as it was, e.g., necessary for the principal-component analysis of the expression profiles of several strains shown in Fig. 1C. Orthologous genes found on the virulence plasmid pYV of strain Y1 and on the chromosome of strain 8081 were excluded.

### Quantitative real-time RT-PCR (qRT-PCR).

qRT-PCR was performed for the validation of RNA-sequencing results on total RNA samples isolated from bacterial cultures grown at 25°C and 37°C to the exponential and stationary phases. For the detection of *ystA* in Y. enterocolitica isolates, total RNA was isolated using an SV total RNA isolation kit (Promega). RNA (35 µg) was treated with 4 units of DNase (Ambion) in a 50-µl reaction mixture. The reaction was then purified using phenol:chloroform:isoamylalcohol. Contamination assessment was performed with PCR and an Agilent 2100 Bioanalyzer (Agilent Technologies). The amount of RNA for each sample was determined using a NanoDrop One Spectrophotometer (Thermo Fisher Scientific). qRT-PCR was performed using a SensiFastNoRox kit (Bioline) with 25 ng/µl of the RNA samples according to the instructions of the manufacturers. qRT-PCR was performed in a Rotor-Gene Q lightcycler system (Qiagen). The primers used for analyzing relative gene expression levels were purchased from Metabion and are listed in Table S1. The *sopB* (validation) and *gyrB* (*ystA* expression) genes were used for normalization. Data analysis was performed with Rotor-Gene Q Series software. Relative gene expression levels were calculated as described earlier (87). Primer efficiencies were determined experimentally using serial dilutions of genomic Y. enterocolitica Y1 and 8081v DNA. The calculated levels of primer efficiency were as follows: for *ystA* (YEY1_01327/YE8081_01824), 2.02; for *sopB* (YEY1_04214/YE8081_04390), 2.21; for *gyrB* (YEY1_00004/YE8081_04289), 2.04; for *ureA* (YEY1_00981/YE8081_00974), 1.98; for *metR* (YEY1_03883/YE8081_00252), 2.00; for *smfA* (YEY1_03315/YE8081_00789), 2.03; for *fimA*-6 (YEY1_03976/YE8081_00164), 2.05; for *glnH* (YEY1_02796/YE8081_02909), 2.13; for *astC* (YEY1_01889/YE8081_02525), 2.03; for *leuO* (YEY1_00693/YE8081_00670), 1.94; for Ysr212, 2.07; for Ysr109, 2.17; for Ysr021, 1.96; for Ysr060, 1.99; for Ysr143, 2.11.

### Analysis of reporter gene expression.

The assay of the β-galactosidase activity of the *lacZ* fusion constructs was performed as described previously (88). The activity was calculated as follows: β-galactosidase activity = OD_420_ * 6,648^−1^ * OD _600_^−1^ * *t* (min)^−1^ * volume (ml)^−1^.

### Data availability.

The complete Y1 genome sequence was deposited in NCBI under accession numbers CP030980 (chromosome YEY1_1) and CP030981 (plasmid YEY1_2). All high-throughput short read data and gene expression quantification information and the DESeq2 result list for all comparisons are deposited at the Gene Expression Omnibus (GEO) database under accession no. GSE119404. A complete list of the TSSs and antisense and *trans*-encoded sRNAs is provided in Data Set S1. The comparative transcriptome analyses are given in Data Sets S2, S3, and S4.
