# Supplementary figures and images for: Comparative Transcriptomic Profiling of Yersinia enterocolitica O:3 and O:8 Reveals Major Expression Differences of Fitness- and Virulence-Relevant Genes Indicating Ecological Separation
Source: mSystems. 2019 Apr 23;4(2):e00239-18. doi: 10.1128/mSystems.00239-18 (PMC6478967; doi:10.1128/mSystems.00239-18)

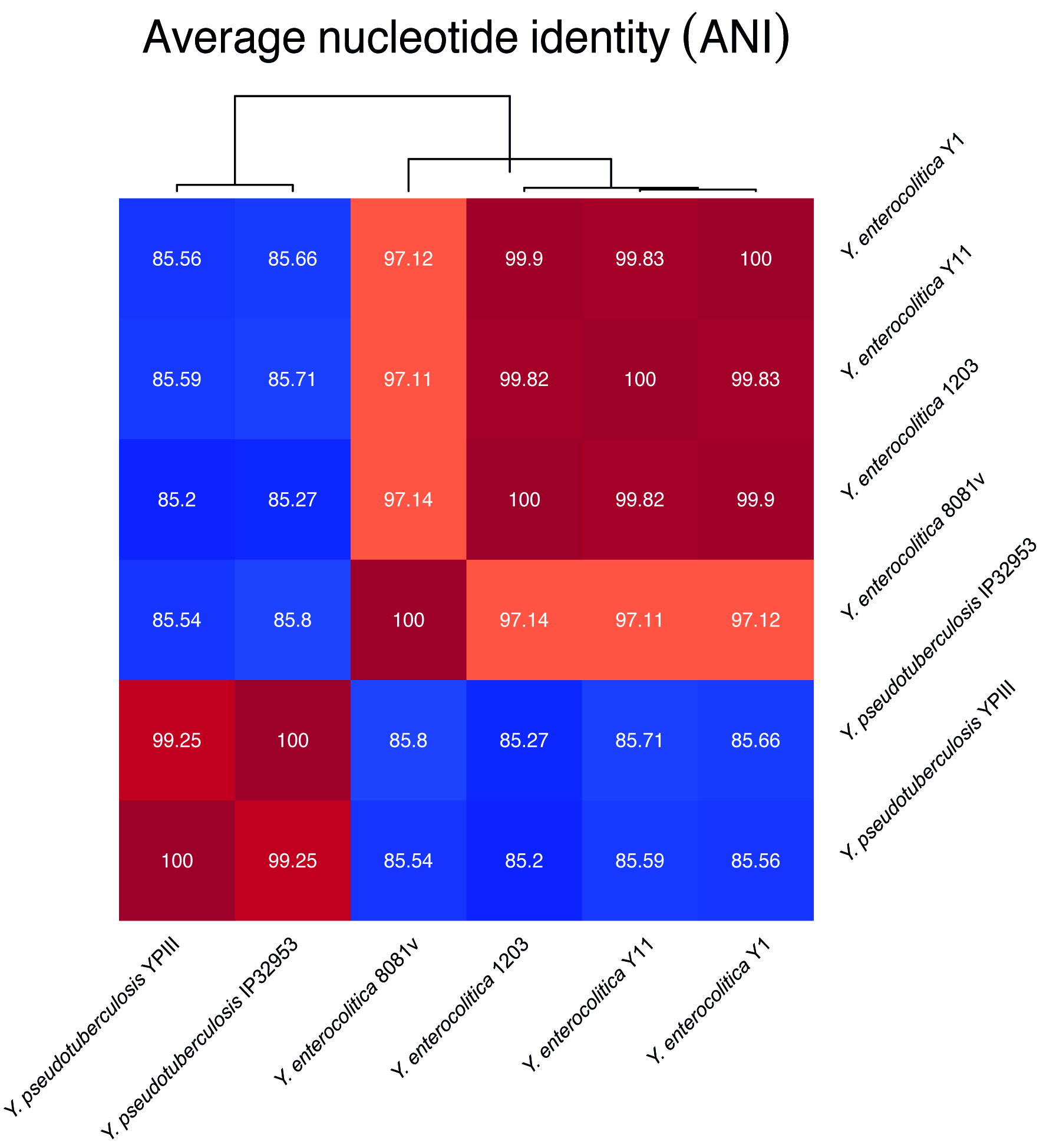

Supplement: FIG S1 [file mSystems.00239-18-sf001.tif]

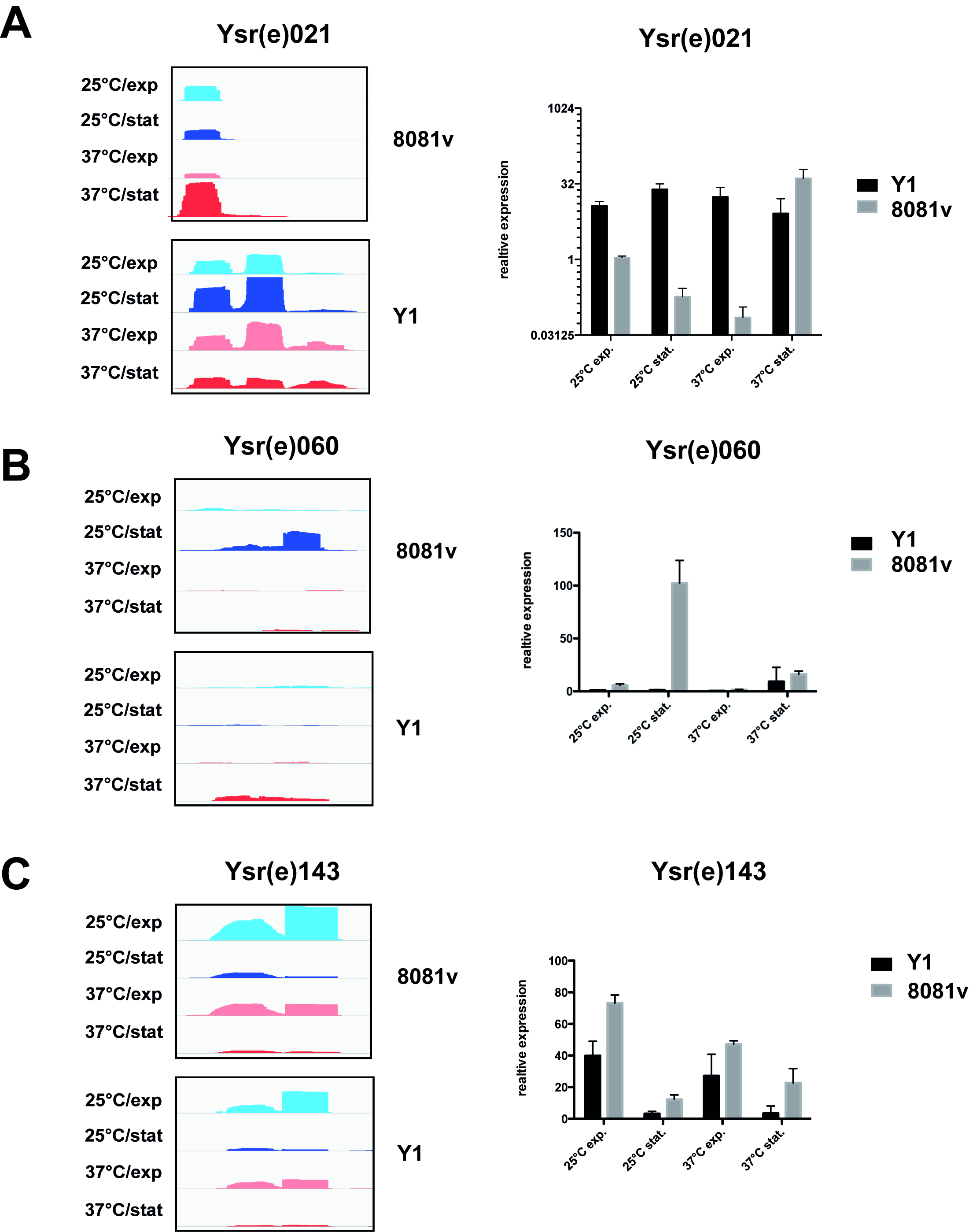

Supplement: FIG S2 [file mSystems.00239-18-sf002.tif]

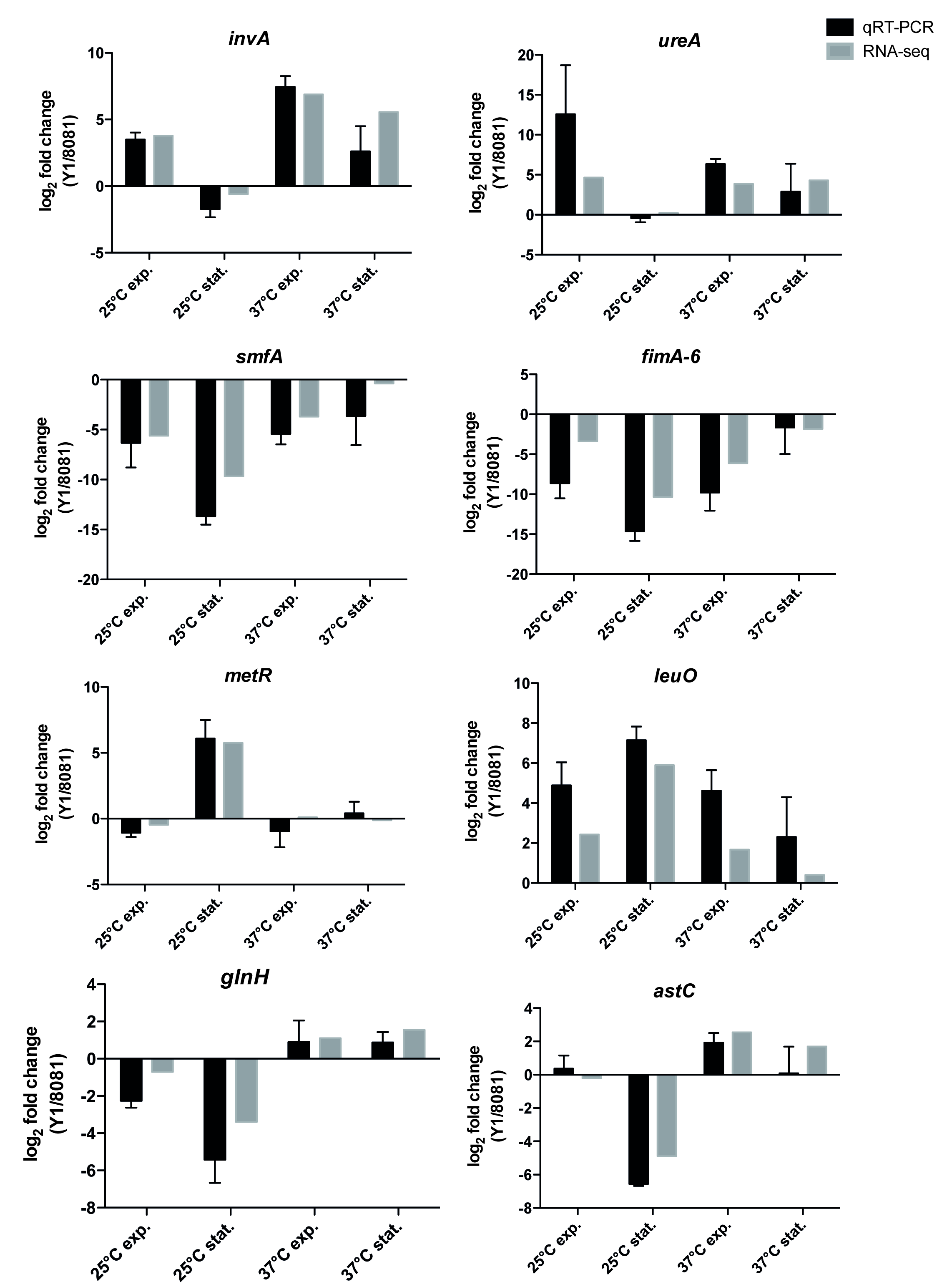

Supplement: FIG S3 [file mSystems.00239-18-sf003.tif]

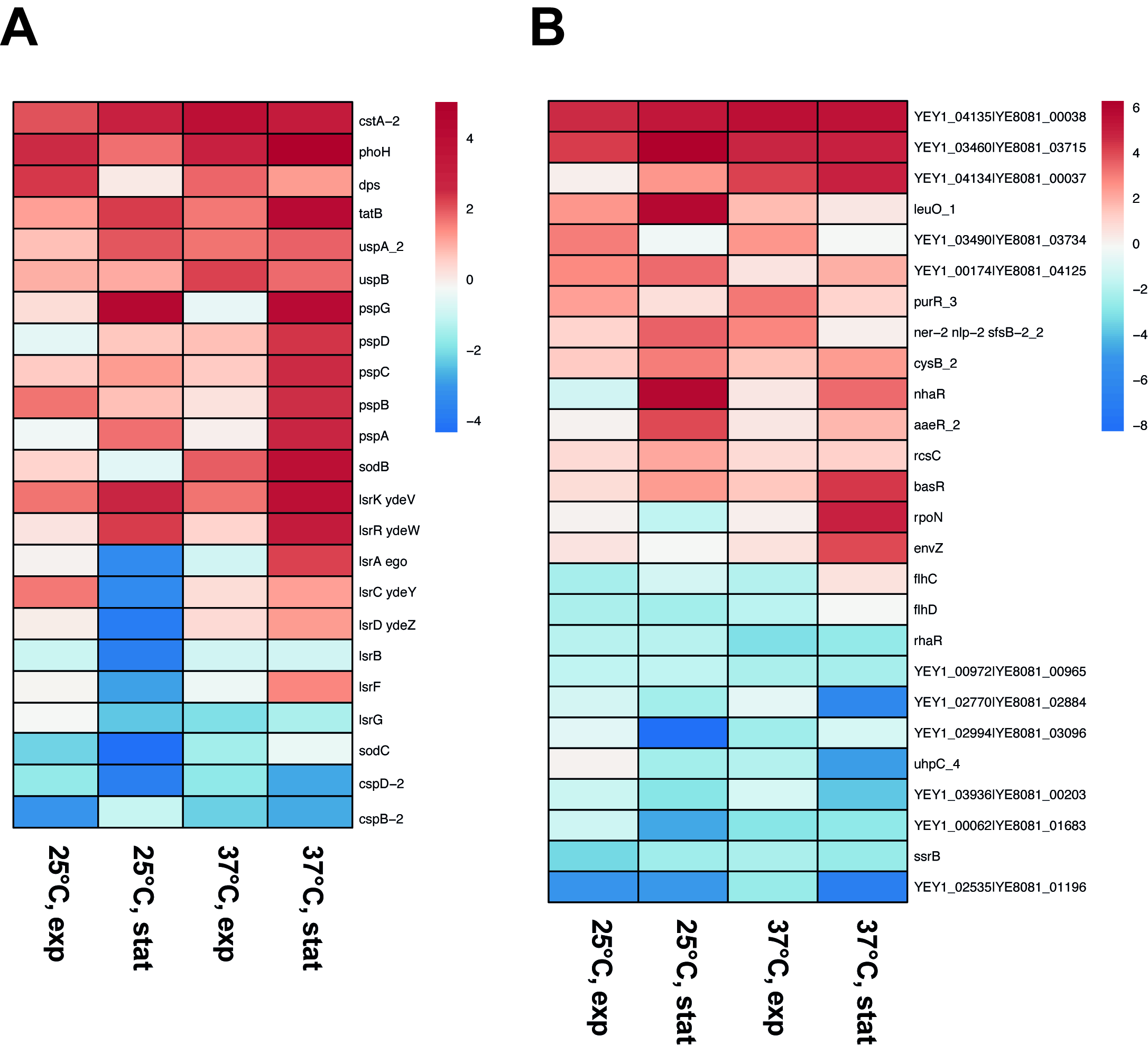

Supplement: FIG S4 [file mSystems.00239-18-sf004.tif]

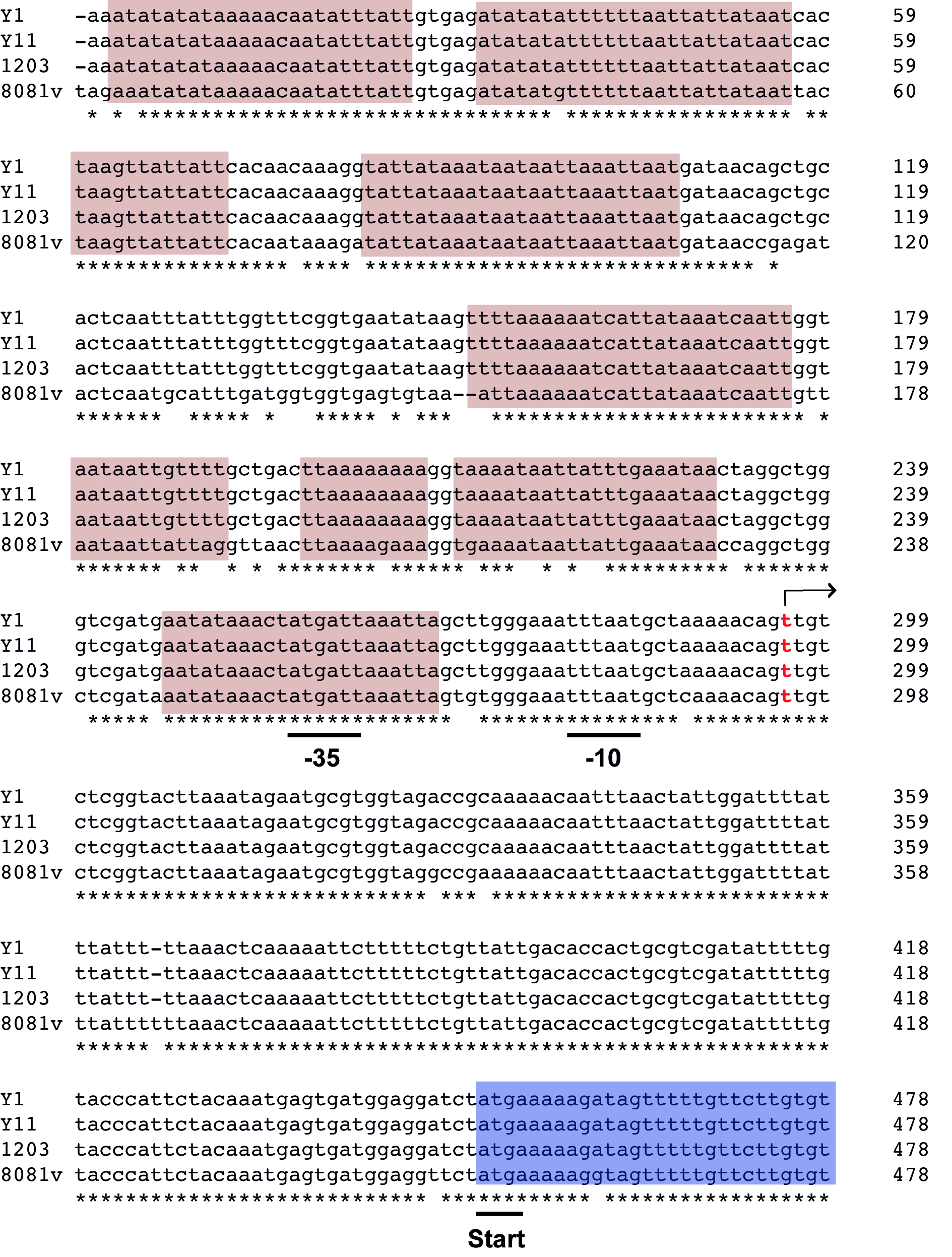

Supplement: FIG S5 [file mSystems.00239-18-sf005.tif]
