# Supplementary material for: Comparative Transcriptomic Profiling of Yersinia enterocolitica O:3 and O:8 Reveals Major Expression Differences of Fitness- and Virulence-Relevant Genes Indicating Ecological Separation
Source: mSystems. 2019 Apr 23;4(2):e00239-18. doi: 10.1128/mSystems.00239-18 (PMC6478967; doi:10.1128/mSystems.00239-18)
Supplement: TABLE S1 [file mSystems.00239-18-st001.docx]

Table S1: Bacterial strains, plasmids and oligonucleotides

**Bacterial Strains**

| **Bacterial Strains** | **Description** | **Source & Reference** |
| --- | --- | --- |
| ***E. coli*** |  |  |
| DH10β | F - *endA*1 *recA*1 *galE*15 *galK*16 *nupG* *rpsL galU deoR* Δ*lacX*74 Φ80*dlacZ*ΔM15 *araD*139Δ(*ara*, *leu*)7697 *mcrA* Δ(*mrr-hsdRMS*-*mcrBC*)λ^-^ | (1) |
| CC118λpir | F Δ(*ara, leu*) 7697 Δ(*lacZ*)74 Δ(*phoA*)20 *araD*139 *galE* *galK thi rpsE rpoB arfE*  *am recA1*, λpir | (2) |
| ***Y. enterocolitica*** |  |  |
| Y1 | O:3, Biotype 4, BfR.Nr. Y1/07, human isolate from feces of a diarrhea patient, 2007 | (3) |
| 8081v | O:8, Biotype 1B, USA | (4) |
| Y34 | O:3, Biotype 4, BfR.Nr. Y34/07, human isolate from feces of a diarrhea patient, 2007 | (3) |
| 08-01985 | O:3, Biotype 4, RKI-Nr. 08-01985, human isolate from feces of a diarrhea patient, 2008 | RKI, F. Uliczka |
| FBI_033 | O:3, Biotype 4, FBI_033 | TiHo, F. Uliczka |
| Y59 | O:3, Biotype 4, BfR.Nr. Y54/07 (Y59) | (3) |
| Y71 | O:3, Biotype 4, BfR.Nr. Y8/08 (Y71) | (3) |
| Y11 | O:3, Biotype 4, Y11, sequenced by DSMZ:13030 | (3) |
| 6471/76 | O:3, 6471/76, wildtype patient isolate | M. Skurnik |
| FBI_03778 | O:3 Biotype 4, FBI_03778 | TiHo, P. Valentin-Weigand |
| C32M1 | O:3, isolate from Europe, A. Mellado | M. Skurnik |
| JH 5700/84 | O:3, isolate from Hamburg, 1984, J. Heesemann | M. Skurnik |
| JH 1131/84 | O:3, isolate from Hamburg, 1984, J. Heesemann | M. Skurnik |
| JD E675 | O:3, human isolate from North America, before 1980, J. Devenish | M. Skurnik |
| S.T.8204 | O:3, isolate from Canada, before 1985, S. Toma | M. Skurnik |
| 80016 | O:3, Biotype 4, human stool isolate, 2006, MaKeRa project, Helsinki, Finland | M. Skurnik |
| 84053 | O:3, Biotype 4, human stool isolate, 2006, MaKeRa project, Helsinki, Finland | M. Skurnik |
| 1870/73 | O:3, isolate from Turku, Finland, 1973 | M. Skurnik |
| 1150/73 | O:3, isolate from Turku, Finland, 1973 | M. Skurnik |
| 37 | O:3, API 0114520, isolate from Oulu, Finland, 1985 | M. Skurnik |
| GKp774 | O:3, isolate from Norway 1975-1985,  G. Kapperud | M. Skurnik |
| GK21603 | O:3, isolate from Norway 1975-1985,  G. Kapperud | M. Skurnik |
| E675 | O:3, isolate from Europe, A. Mellado | M. Skurnik |
| RBC36M3 | O:3, isolate, 1983, B. Perry | M. Skurnik |
| 1203 | O:3, Biotype 4 | A. McNally |
| Y18 | O:8, Biotype 1B, BfR.Nr.79/90 (Y18) | (3) |
| Y19 | O:8, Biotype 1B, BfR.Nr. Ye12 (Y19) | (3) |
| Y49 | O:8, Biotype 1B, BfR.Nr. 39/91 (Y49) | (3) |
| 07-00628 | O:9, Biotype 3, RKI-Nr. 07-00628 | RKI, F. Uliczka |
| Y24 | O:9, Biotype 3, BfR.Nr. 2012/79 (Y24) | (3) |
| Y31 | O:9, Biotype 3, BfR.Nr. 495/88 (Y31) | (3) |
| Y48 | O:9, Biotype 3, BfR.Nr. 7/91 (Y48) | (3) |
| 21202 | O:9, Biotype 2 | A. McNally |
| 5603 | O:9, Biotype 3 | A. McNally |
| YE12 | Y1 Δ*rovA* | (3) |

**Plasmids**

| **Plasmid** | **Description** | **Source & Reference** |
| --- | --- | --- |
| pAKH71 | pACYC184, *ymoA*^+^, Cm^R^ | (5) |
| pAKH74 | pACYC184, *hns*^+^, Cm^R^ | (6) |
| pHT109 | pZA31, *rovA^+^,* Cm^R^ | H. Tran-Winkler |
| pFU55 | Cloning vector for *lacZ* (translational fusions), pSC101, *Kan^R^ | (7) |
| pCS68 | pFU55, *ystA*(O:8)-*lacZ*, , fusion of P*_ystA_* (position --582 to +11) to *lacZ,* pSC101, *Kan^R^ | This study |
| pCS63 | pFU55, *ystA*(O:3)-*lacZ*, fusion of P*_ystA_* (position -203 to +11) to *lacZ,* pSC101, *Kan^R^ | This study |
| pCS68 | pFU55, *ystA*(O:8)-*lacZ*, fusion of P*_ystA_* (position -389 to +11) to *lacZ,* pSC101, *Kan^R^ | This study |
| pCS70 | pFU55, *ystA*(O:8)-*lacZ*, fusion of P*_ystA_* (position -203 to +11) to *lacZ,* pSC101, *Kan^R^ | This study |
| pCS71 | pFU55, *ystA*(O:3)-*lacZ*, fusion of P*_ystA_* (position - 582 to +11) to *lacZ,* pSC101, *Kan^R^ | This study |
| pCS72 | pFU55, *ystA*(O:3)-*lacZ*, fusion of P*_ystA_* (position - 389 to +11) to *lacZ,* pSC101, *Kan^R^ | This study |

| **Oligonucleotides** | | |
| --- | --- | --- |
| **Name** | **Sequence (5’- 3’ orientation)** | **Description/Target Sequence** |
| ***Oligonucleotides used for cloning*** | | |
| VIII9 | GCGGCG**CTCGAG**TCTGGTAACGAAAAGAG | forward primer for *ystA* upstream region (-582) of *Y. enterocolitica* O:3 and O:8 harboring a *Xho*I site |
| VIII009 | GCGGCGCCTCGAGTCTGGTAACGAAGAG | cloning of *ystA* 5’UTR (-582 bp) of *Y. enterocolitica* O:3 and O:8 harboring a *Xho*I site |
| VIII010 | GCGGCGCCTCGAGAGTTATTATTCACAACAAAGG | forward primer for *ystA* (O:3) upstream region (-389) harboring a *Xho*I site |
| VIII011 | GCGGCGCCTCGAGAGATATAAACTATGATTAAATTAGC | forward primer for *ystA* (O:3) upstream region (-203) harboring *Xho*I site |
| VIII014 | GCGGCGCCTCGAGAGTTATTATTCACAATAAAG | forward primer for *ystA* (O:8) upstream region (-389) harboring a *Xho*I site |
| VIII015 | GCGGCGCCTCGAGATATAAACTATGATTAAATTAGTG | forward primer for *ystA* (O:8) upstream region (-203) harboring *Xho*I site |
| VIII016 | GCGGCGCGCTAGCCTATCTTTTTCATAGATCCTCC | reverse primer *ystA* (O:3) coding region (+11) harboring *Nhe*I site |
| VIII019 | GCGGCGCGCTAGCTACCTTTTTCATAGAACCTCC | reverse primer *ystA* (O:8) coding region +11) harboring *Nhe*I site |
| ***Oligonucleotides used for qRT-PCR*** | | |
| VIII22 | GATAGTTTTTGTTCTTGTG | forward primer qRT for *ystA*, *Y. enterocolitica* O:3 |
| VIII23 | CTAGCAGCCAGCACAC | forward primer qRT for *ystA*, *Y. enterocolitica* O:8 |
| VIII40 | CTAGCAACCCGCACAG | reverse primer qRT for *ystA*, *Y. enterocolitica* O:3 and O:8 |
| III95 | ACCCCGCTGAACATAATGAG | forward primer qRT for *invA* of *Y. enterocolitica* O:3 and O:8 |
| III96 | TGCCGCGTCATTTACCATTG | reverse primer qRT for *invA* of *Y. enterocolitica* O:3 and O:8 |
| III182 | CCGGTGGTTTGCACGGCGT | forward primer qRT for *gyrB* of *Y. enterocolitica* O:3 and O:8 |
| III183 | CACCACTTTCAATGGTGCC | reverse primer qRT for *gyrB* of *Y. enterocolitica* O:3 and O:8 |
| VIII41 | AACGCAAAGCGCGTGGC | forward primer qRT for *ureA* of *Y. enterocolitica* O:3 and O:8 |
| VIII42 | GGAATCAGATCAGCCACC | reverse primer qRT for *ureA* of *Y. enterocolitica* O:3 and O:8 |
| VIII57 | TGAAACACTTACGCACCC | forward primer qRT for *metR* of *Y. enterocolitica* O:3 and O:8 |
| VIII58 | GTTGTGAAACGTAACGGC | reverse primer qRT for *metR* of *Y. enterocolitica* O:3 and O:8 |
| VIII83 | CAATTCAATATAAATGAATTTGG | forward primer qRT for *smfA* of *Y. enterocolitica* O:3 and O:8 |
| VIII95 | TGCTGGTCTGGTATTAGG | forward primer qRT for *invA* of *Y. enterocolitica* O:3 and O:8 |
| VIII96 | CGCCATTTTGCAGTGCC | reverse primer qRT for *smfA* of *Y. enterocolitica* O:3 and O:8 |
| VIII101 | GTAGCAATGGCAGCAAGC | forward primer qRT for *fimA-6* of *Y. enterocolitica* O:3 and O:8 |
| VIII102 | AACCTGATACACCCAGAG | reverse primer qRT for *fimA-6* of *Y. enterocolitica* O:3 and O:8 |
| VIII105 | AAGAACTGATTGTTGCCAC | forward primer qRT for *glnH* of *Y. enterocolitica* O:3 and O:8 |
| VIII106 | CCAGTGCCAGATCCACG | reverse primer qRT for *glnH* of *Y. enterocolitica* O:3 and O:8 |
| VIII121 | GTGGTACGAGGCGAAGG | forward primer qRT for *astC* of *Y. enterocolitica* O:3 and O:8 |
| VIII122 | GCCAGTCGCAACACCGG | reverse primer qRT for *astC* of *Y. enterocolitica* O:3 and O:8 |
| VIII123 | AACGATGTTCATTTACGC | forward primer qRT for *leuO* of *Y. enterocolitica* O:3 and O:8 |
| VIII124 | ACCTCGACCATAACGCAC | reverse primer qRT for *leuO* of *Y. enterocolitica* O:3 and O:8 |
| VIII441 | TATCTATCTATCTATCTATCTATC | reverse primer qRT-PCR Ysr(e)021 in YeO:3 |
| VIII442 | ATTATGGATTATTGTTCTC | forward primer qRT-PCR Ysr(e)021 in YeO:8 |
| VIII454 | CCAAGAGTTTTCTGGCAC | forward primer qRT-PCR Ysr(e)060 |
| VIII455 | GAACTTAATTTCTATTGGCG | reverse primer qRT-PCR Ysr(e)060 |
| VIII469 | TGTTATGCAATAGTCATGC | forward primer qRT-PCR Ysr(e)109 |
| VIII470 | AAACCCCGGCGAAAACC | reverse primer qRT-PCR Ysr(e)109 |
| VIII480 | TCGACACGGCGCTGTG | forward primer qRT-PCR Ysr(e)143 in YeO:8 |
| VIII481 | CGCGGGAGGCAGATAATACG | reverse primer qRT-PCR Ysr(e)143 |
| VIII493 | ATGGCGGCTGAGTGCTG | forward primer qRT-PCR Ysr(e)212 |
| VIII494 | GTTCGTCATGCGCCACC | reverse primer qRT-PCR Ysr(e)212 |

BfR: Federal Agency for Risk Assessment Germany

RKI: Robert Koch Institute, Germany

TiHo: University of Veterinary Medicine Hanover, Germany

**References:**

1. **Durfee T, Nelson R, Baldwin S, Plunkett G, 3rd, Burland V, Mau B, Petrosino JF, Qin X, Muzny DM, Ayele M, Gibbs RA, Csorgo B, Posfai G, Weinstock GM, Blattner FR.** 2008. The complete genome sequence of *Escherichia coli* DH10B: insights into the biology of a laboratory workhorse. J Bacteriol **190:**2597-2606.

2. **Manoil C, Beckwith J.** 1986. A genetic approach to analyzing membrane protein topology. Science **233:**1403-1408.

3. **Uliczka F, Pisano F, Schaake J, Stolz T, Rohde M, Fruth A, Strauch E, Skurnik M, Batzilla J, Rakin A, Heesemann J, Dersch P.** 2011. Unique cell adhesion and invasion properties of *Yersinia enterocolitica* O:3, the most frequent cause of human Yersiniosis. PLoS Pathog **7:**e1002117.

4. **Portnoy DA, Moseley SL, Falkow S.** 1981. Characterization of plasmids and plasmid-associated determinants of *Yersinia enterocolitica* pathogenesis. Infect Immun **31:**775-782.

5. **Böhme K, Steinmann R, Kortmann J, Seekircher S, Heroven AK, Berger E, Pisano F, Thiermann T, Wolf-Watz H, Narberhaus F, Dersch P.** 2012. Concerted actions of a thermo-labile regulator and a unique intergenic RNA thermosensor control *Yersinia* virulence. PLoS Pathog **8:**e1002518.

6. **Heroven A, Nagel G, Tran HJ, Parr S, Dersch P.** 2004. RovA is autoregulated and antagonizes H-NS-mediated silencing of invasin and *rovA* expression in *Yersinia pseudotuberculosis*. Mol Microbiol **53:**871-888.

7. **Uliczka F, Pisano F, Kochut A, Opitz W, Herbst K, Stolz T, Dersch P.** 2011. Monitoring of gene expression in bacteria during infections using an adaptable set of bioluminescent, fluorescent and colorigenic fusion vectors. PLoS One **6:**e20425.
